# dynConfiR: An R package for sequential sampling models of decision confidence

**DOI:** 10.3758/s13428-026-03013-0

**Published:** 2026-05-08

**Authors:** Sebastian Hellmann, Michael Zehetleitner, Manuel Rausch

**Affiliations:** 1 Chair of Behavioral Research Methods, TUM School of Management, Munich, Germany; 2 Philosophical-pedagogical Faculty, Catholic University of Eichstätt-Ingolstadt, Eichstätt, Germany; 3 Faculty of Society and Economics, Rhine-Waal University of Applied Sciences, Cleves, Germany; 4 Department of Psychology, University of Klagenfurt, Klagenfurt, Austria

**Keywords:** R package, Cognitive modeling, Confidence, Decision-making, Drift-diffusion models, Sequential sampling models

## Abstract

The modeling of response times using sequential sampling models has a long history. Because choices, confidence judgments, and reaction times are closely linked in perceptual decisions, it seems only natural to simultaneously model these three outcome variables of a decision. In the package dynConfiR, we implemented various sequential sampling models of choice, response time, and decision confidence in R. This paper gives an overview of the package, which provides probability density functions as well as high-level functions for fitting parameters to empirical data, prediction of reaction time and response distributions, and simulation of artificial data sets. We describe the mathematical specifications of the implemented models and provide detailed descriptions of the implemented likelihood functions. In addition, we outline the workflow for applying the model to empirical data step-by-step: data preprocessing, model fitting, model prediction, quantitative model comparison, and visual assessment of model predictions. Finally, we present results from parameter and model recovery analyses and assess the precision of probability density calculations, illustrating the robustness of the implemented computations. Offering intuitive usability and high flexibility, the package is targeted at researchers in the fields of decision-making and confidence and does not require expert-level programming skills.

**Supplementary Information:**

The online version contains supplementary material available at 10.3758/s13428-026-03013-0.

## Introduction

Recently, confidence has gained increasing research interest in the field of cognitive computational modeling (e.g., Aitchison et al., 2015; Rausch et al., 2018; Kiani et al., 2014; Desender & Donner, 2021; Moran et al., 2015; Adler, 2018; Hellmann et al., 2024, 2023; Maniscalco, 2016; Maniscalco et al., 2016; Pleskac, 2010; Ratcliff & Starns, 2013, 2009; Zawadzka et al., 2017). Many experimental tasks and everyday decisions include uncertainty, so the decision-maker cannot be entirely sure whether their decision was correct. The resulting degree of belief in the correctness of one’s decision is referred to as confidence (Pouget et al., 2016). Because confidence is also relevant in everyday behavior and communication, for example, when driving in a foggy environment or making difficult medical diagnoses, it is essential to understand how confidence arises from the decision process.

Many models of confidence are based on traditional signal detection theory (SDT, Green, 1966). We refer to these models as static models, as they do not explain the single-trial dynamics of a decision but assume that the decision is made by comparing a single random variable against a criterion. Traditional SDT models have been extended to account for confidence judgments, for example, by introducing additional criteria, information gain, or noise in the confidence judgments (Adler, 2018; Mamassian & de Gardelle, 2022; Rausch et al., 2018, 2023; Shekhar & Rahnev, 2021, 2024). Static confidence models have proven successful in accounting for the relationship between task difficulty and confidence and have been useful for explaining discrepancies between confidence judgments and actual accuracy. Although response times may be incorporated into these models to account for the dynamic properties of the generation of evidence on which the decisions are based, e.g., by scaling both the magnitude and noise with the response time, they do not provide an explanation for how response times are generated as a dependent variable. For this reason, static models may be applied to interrogation tasks (Bogacz et al., 2006), in which the experimenter externally controls how long evidence may be accumulated, but cannot account for the empirical patterns like the negative relationship between discriminability and response times that are commonly observed in free response tasks, in which subjects themselves determine when to make a decision. In addition, confidence is also closely related to decision time in many free response tasks (Hellmann et al., 2024; Kiani et al., 2014; Rahnev et al., 2020; Vickers et al., 1985). In contrast to static models, dynamic models explain the generation of response time distributions and may thus provide insight into the causal relationship between task difficulty, decision time, and confidence. Many dynamic models of decision-making assume a sequential sampling process, that is, evidence is sampled from a noisy distribution repeatedly over time, and an internal decision variable is updated until a particular stopping rule is met and the decision is triggered (Ratcliff et al., 2004). It should be noted that most sequential sampling models of decision making are based on signal detection principles and thus the term dynamic signal detection theory is occasionally used for random walk models (Pleskac, 2010; Smith, 2000). However, dynamic decision models exist that assume linear and deterministic dynamics for the accumulation process (Brown & Heathcote, 2005; Brown, 2008; Reynolds et al., 2020).

A prominent example of a computational model in the field of decision-making research is the drift-diffusion model (DDM, Stone, 1960; Link, 1975; Link & Heath, 1975). Since it was initially formulated, it was extended by including additional parameters and applied in various experimental tasks (Ratcliff, 1978; Ratcliff et al., 2004).

However, the DDM, in its original conception, does not account for confidence judgments. In two previous studies, we compared different confidence models based on two important prototypes of dynamic models of decision-making, the DDM and the race of accumulators (Hellmann et al., 2023, 2024). We demonstrated that fitting the joint distribution of choice, response time, and confidence is useful for testing computational models of decision making and is more desirable than fitting summary statistics of the data.

The presented package includes functions to fit response times and confidence judgments in binary choice tasks based on the following models: the drift-diffusion confidence model (DDConf, Hellmann et al., 2023; Ratcliff, 1978), the two-stage dynamic signal detection model (2DSD, Pleskac, 2010), the dynamical weighted evidence and visibility model (dynWEV, Hellmann et al., 2023), the dynamical visibility, time, and evidence model (dynaViTE, Hellmann et al., 2024), several versions of race models (Moreno-Bote, 2010), and the multiple-threshold correlated log-normal race model (MTLNR, Reynolds et al., 2020). The models are explained in more detail in the next section.

Due to their mathematical complexity, dynamic models of decision-making and confidence are challenging to implement. By providing the **dynConfiR** package, we aim to remove the hurdle of implementing likelihood functions and fitting procedures to facilitate the application of confidence models for research questions in psychology and cognitive neuroscience.

### Alternative software

Software already exists to analyze response time data for decision models, mainly in the context of the DDM. Some examples are the **R** packages rtdists (Singmann et al., 2020), which offers probability distribution and simulation functions for the seven-parameter DDM and the linear ballistic accumulator model (Brown, 2008), and RWiener (Wabersich, 2014a), which provides an implementation of the four-parameter DDM with functions for parameter fitting. Fast-dm (Voss & Voss, 2007) is a stand-alone command line tool for fitting the seven-parameter DDM to empirical data. This tool also allows fitting the model with arbitrary parameters varying across different conditions. The Diffusion Model Visualizer is a graphical user interface that allows users to explore how the parameters of the seven-parameter DDM affect response time distributions, but it does not provide functionality for fitting parameters to empirical data or computing likelihoods (Alexandrowicz, 2020).

The Python toolbox HDDM allows for hierarchical parameter estimation of DDM parameters (Wiecki et al., 2013). Similarly, the EMC2 is an R package that allows for the Bayesian estimation of parameters in various evidence accumulation models to choice and response time data (Stevenson et al., 2024). These packages are immensely flexible when it comes to the specification of the hypothesized effect of experimental manipulations and other predictors on model parameters. In that sense, confidence judgments could be incorporated into the analysis as an additional predictor, e.g., for guiding learning (Drugowitsch et al., 2019) or influence the decision-threshold in upcoming trials (Desender et al., 2019). However, these tools do not allow scientists to easily include confidence judgments as an additional dependent variable in the computational models.

**Table 1:** Comparison of features of the **dynConfiR** to other existing software to fit dynamic models of decision making

		Dependent variables		User-accessible functions		
Software	Programming language	Response times	Confidence judgments	Density functions	Fitting functions	Statistical paradigm
rtdists	R	$\checkmark$	✗	$\checkmark$	✗	frequentist
RWiener	R	$\checkmark$	✗	$\checkmark$	$\checkmark$	frequentist
Fast-dm	stand-alone command line tool	$\checkmark$	✗	✗	$\checkmark$	frequentist
HDDM	Python	$\checkmark$	✗	$\checkmark$	$\checkmark$	Bayesian
EMC2	R	$\checkmark$	✗	✗	$\checkmark$	Bayesian
wiener module	JAGS	$\checkmark$	✗	–	–	Bayesian
lpdf_wiener	Stan	$\checkmark$	✗	–	–	Bayesian
statConfR	R	✗	$\checkmark$	✗	$\checkmark$	frequentist
ReMeta	Python	✗	$\checkmark$	✗	$\checkmark$	frequentist
cfc	Matlab	✗	$\checkmark$	✗	$\checkmark$	frequentist
dynConfiR	R	$\checkmark$	$\checkmark$	$\checkmark$	$\checkmark$	frequentist

$\checkmark$: available; ✗: not available; –: not applicable

In addition, the Wiener module for JAGS (Wabersich & Vandekerckhove, 2014b) and Stan allow researchers to easily incorporate the DDM in more complex probabilistic models (see e.g. Fontanesi et al., 2019, for an application in reinforcement learning). Recently, the seven-parameter DDM was implemented in Stan (Henrich et al., 2024). Using JAGS or Stan, scientists could also build more complex models to account for confidence data; however, this requires at least some knowledge in MCMC sampling and knowledge of either software tool. In addition, to include other models that are not based on the DDM, such as race models, researchers would have to write their own likelihood function extensions.

As a summary, there are a lot of alternatives when it comes to the modeling of choices and response times. These alternatives are already very popular and provide a high degree of flexibility and some allow for the hierarchical estimation of model parameters, which is beneficial if only a limited number of trials are available for each subject.

For fitting confidence data, the statConfR package (Rausch et al., 2025) allows for parameter fitting in the context of static confidence models and the computation of popular measures of metacognitive performance such as meta-d$'$ (Maniscalco & Lau, 2012). The ReMeta toolbox is a Python library that facilitates the estimation of parameters of static confidence models with a high degree of flexibility in specifying the data-generating process (Guggenmos, 2022). Finally, Mamassian and de Gardelle (2022) provided a Matlab toolbox to fit confidence judgments in the so-called confidence forced-choice paradigm. However, these software packages are based on signal-detection models and cannot account for response times.

Table 1 summarizes and compares available software to the **dynConfiR** package. Only very few software tools currently exist that implement sequential sampling models of confidence. The DMC software includes an implementation of the multiple-threshold linear ballistic accumulator model and the MTLNR (Heathcote et al., 2019; Reynolds et al., 2020). Unfortunately, at the time of writing, the DMC software is no longer actively maintained, and comprehensive documentation for applying these models is not available. Instead, the authors of DMC are planning to extend the EMC2 software to include confidence models in a future release (A. Heathcote, personal communication, September 18, 2025). **dynConfiR** provides implementations of the joint distribution of choice, response time, and confidence judgment for several sequential sampling models of decision confidence together with wrapper functions to compute the likelihood for a whole data set and a given set of parameters. This enables advanced users to define custom likelihood functions tailored to their specific experiments. Particularly, researchers can implement their own mapping between stimulus properties and drift rates in two-alternative force-choice tasks, and incorporate attentional discounts when eye-tracking data are available (Krajbich et al., 2010).

In addition, the package includes functions for maximum-likelihood parameter estimation and for predicting or simulating distributions, which facilitates the use by less-advanced users who want to focus on the application of the models to their data. Functions are written to provide an intuitive and straightforward way to implement the whole workflow of model fitting and comparison with only a few lines of code, while still providing possibilities for customization, for example, fixing parameters that should not be fitted.

### Scope and limitations of the package

Although the dynConfiR package implements various computational models covering a broad range of concepts (e.g., models with post-decisional accumulation time, race models, and drift-diffusion models) and the fitting procedure is user-friendly, there are some limitations concerning both the scope and the usability of the package that we will describe below.

First, we restricted the implementation of models to confidence models for which the likelihood of the joint distribution of choices, response times, and confidence judgments is mathematically tractable. This means that the models do not capture several aspects and variations of the models discussed in the literature, e.g., collapsing boundaries and leakage. All models assume time-constant boundaries and stationary drift rates that are independent of the process’s current state. In addition, the race models do not allow for inhibition. Time-collapsing boundaries are discussed in the literature to implement time costs in the diffusion process, and leakage and inhibition are referred to as neurally plausible mechanisms of accumulation processes (Tajima & Drugowitsch, 2016; Usher & McClelland, 2001). In addition, several dynamic confidence models like RTCON and RTCON2 (Ratcliff & Starns, 2009, 2013) and the bounded accumulation model by Kiani et al. (2014) are not implemented in the package. As new computational models of confidence judgments keep being proposed, we plan to extend the range of models in the future with other models. In addition, we encourage contributions to the package by other researchers (see the GitHub page of the package).

Second, although some models assume post-decisional evidence accumulation, no model implemented to date accounts for confidence response times in paradigms with subsequent confidence reports. In such situations, one has to rely on the observed confidence response times to inform the post-decisional accumulation period (see Hellmann et al., 2024). Some proposed models account for confidence response times but are not yet implemented in dynConfiR (Herregods et al., 2023; Moran et al., 2015). Importantly, the models assuming post-decisional evidence accumulation accounted for response times and confidence judgments in paradigms with simultaneous choice and confidence reports and even outperformed the alternative models that did not include post-decisional accumulation (Hellmann et al., 2023).

Third, although we restrict the package to models with a feasible likelihood, the parameter-fitting procedure requires substantial computational time. Because the implemented maximum likelihood procedure for parameter fitting requires the computation of the joint distribution on a trial-level for each iteration, even with the effective finite sum approximations of the response time density in the diffusion-based models, the computational effort is considerable (see also the Sections “Model recovery analysis” and “Parameter recovery analysis”, where we report the computation times for the likelihood evaluation and parameter fitting).

Finally, some conceptual limitations come with the maximum likelihood estimation technique used for parameter fitting. Unlike in Bayesian estimation methods, the estimated parameters are only point estimates, and there is no measure for the uncertainty of the estimation. There is no implemented method to report confidence intervals for the parameters because the dependency among them (e.g., confidence thresholds need to be ordered) makes the standard procedures for computing confidence intervals difficult. The information criteria used for assessing the goodness-of-fit of the models are also based on the maximum likelihood estimation and do not account for the functional complexity of the models (Myung, 2000). However, the good model identification presented in this article (see Section “Model recovery analysis”) indicates that using BIC for model comparison generally has a low level of mis-identifications when the models are fitted to empirical data.

### Structure of the present paper

In this paper, we first present the confidence models included in the package, explain all parameters, and provide mathematical definitions. The second section provides details about the functionalities of the package. Based on the implemented functionalities, we propose workflows for different use cases and show possibilities for individual settings in the analyses. In addition, we showcase the suggested workflow for model comparison using an empirical data set. The sections “Parameter recovery analysis”, “Precision analysis”, and “Model recovery analysis” contain results from simulation studies examining the performance of the package. All analyses scripts and data sets used in this paper are available at https://github.com/SeHellmann/dynConfiR_Paper.

## Sequential sampling confidence models

In this section, we describe the sequential sampling models included in **dynConfiR** in detail. Most dynamic models of decision-making share the idea that a decision is not based on a single sample, as in SDT. In contrast, sequential sampling models describe decisions as processes in which evidence is repeatedly sampled and accumulated over time (Ratcliff et al., 2004). Starting from a discrete-time perspective and normally distributed samples, reducing the time step size leads to a continuous Wiener process describing the accumulation of evidence. The Wiener process may be interpreted as the stochastic process equivalent to the Gaussian distribution. This is because the functional invariance principle states that, accordingly, scaled partial sum processes, which formalize the idea of sequentially sampling and integrating evidence mathematically, converge to a Wiener process in the limit of small time steps (Klenke, 2013). However, there are dynamic models of decision-making that explicitly use other processes, like the Poisson counter model (LaBerge, 1994) or the leaky competing accumulator model (Usher & McClelland, 2001). The accumulation of evidence continues until enough information in favor of one alternative is available, formalized by a stopping criterion. When the stopping criterion is met, a choice is triggered for the alternative favored by the accumulated evidence. All models included in **dynConfiR** assume that stopping criteria take the form of time-constant absorbing boundaries. Alternative models that suggest collapsing boundaries, which approach the starting point over time (Drugowitsch et al., 2012; Milosavljevic et al., 2010; Tajima & Drugowitsch, 2016), are not included in the package. Sequential sampling models of decision-making provide explanations for the correlation between discriminability and reaction time and the speed–accuracy trade-off (Lerche et al., 2019; Ratcliff & Rouder, 1998). The dynConfiR package features two classes of dynamic confidence models. The first class of models is based on the DDM, which assumes a single accumulation process representing evidence in favor of one choice alternative over the other. The second class of models is race models, which assume multiple accumulation processes, each representing one choice alternative. Among the race models, the MTLNR is treated in an independent section because it does not use Wiener processes but simplifies the accumulation process to a ballistic accumulation without noise (Brown & Heathcote, 2005). In the sections that follow, we first describe the most general model of the first class, the dynamical visibility, time, and evidence model (dynaViTE), and how the other models of this class, DDConf, 2DSD, and dynWEV, are special cases of dynaViTE. We will then describe the second class of models.

### Drift-diffusion-based confidence models

We first present the decision mechanism that is the basis for the first class of models. In the DDM, the decision process is described as a Wiener process, which is bounded by two time-constant thresholds 0 and *a*. The process *X* starts at the starting point *X*(0). The relative starting position between the two thresholds, i.e. *X*(0)/*a*, follows a uniform distribution around the parameter *z* with range $s_z$, formally $X(0)/a \sim {\text {Unif}}[z-s_z/2, z+s_z/2]$. The process then evolves with a drift of $\mu$, which is normally distributed around $\nu$ with standard deviation $s_\nu$. The diffusion constant is denoted as *s*. When the process first hits either the lower or the upper threshold, a decision is triggered. Decision time is thus defined as

$$\begin{aligned} T_{Dec}=min\{t| X(t) \in \{0,a\}\}. \end{aligned}$$

The choice response *R* is -1 if the lower threshold was hit, i.e., if $X(T_{Dec})=0$, and it is +1, otherwise. Correspondingly, in discrimination tasks, the sign of the mean drift rate reflects the true stimulus identity, $S={\text {sig}}(\nu )$, while its magnitude is determined by experimental manipulations in task difficulty (see Sect. Fitting confidence models to experimental data).

#### Dynamical visibility, time, end evidence model (dynaViTE)

The dynaViTE model assumes that the decision process *X* continues after it reaches one of the two thresholds. This accumulation continues for a fixed period of time, which is represented by the parameter $\tau$. In addition, dynaViTE postulates a second process evolving in parallel to the decision process. The second process is denoted as the visibility process *V* and is again a Wiener process, which always starts as 0. Its drift rate is subject to noise similar to the drift rate in the decision process. More precisely, the visibility drift is normally distributed with mean visibility drift $\mu _V$ and standard deviation $\sigma _V$. The diffusion constant of the visibility process is represented by the parameter $s_V$. Importantly, only one parameter in dynaViTE can be fixed to scale the other parameters without affecting model predictions. That is, if the diffusion constant of the decision process *s* is fixed, then $s_V$ cannot be fixed as well without restricting the model.

The psychological interpretation of the two processes is as follows: While the decision process accumulates evidence about the identity of the stimulus, i.e., whether it belongs to the class representing the upper or lower threshold, respectively, the visibility process accrues evidence about stimulus features that are indicative of task difficulty but not informative for the stimulus identity. In visual discrimination tasks – for which dynaViTE was originally proposed – visibility may be task-irrelevant stimulus features like brightness, shape, presentation time, or contour. Importantly, the model is applicable to a wide range of binary perceptual decision tasks, and the concept of visibility may more generally be interpreted as reliability. We retain the term visibility to remain consistent with the existing literature, in which the model was originally developed and applied to visual discrimination tasks (Hellmann et al., 2023, 2024).

Confidence is then a function of the accumulated decision evidence ($X(T_{Dec}+\tau )-az$), visibility evidence ($V(T_{Dec}+ \tau )$), and accumulation time ($T_{Dec}+\tau$). After the post-decisional accumulation period, accumulated evidence in the two processes is combined in a weighted sum and divided by a power of accumulation time to form an internal confidence variable

1 $$\begin{aligned} c_{dynaViTE}=\frac{wR(X(T_{Dec}+ \tau )-az) + (1-w)V(T_{Dec}+\tau )}{(T_{Dec}+ \tau )^\lambda }, \end{aligned}$$

in which the parameter *w* controls the weight on decision evidence compared to visibility evidence, and $\lambda$ controls the penalty of accumulation time on confidence. The factor *R* in the numerator of Eq. 1 leads to a positive scaling of choice congruent evidence in the case when the choice is $R=-1$ (i.e., the lower threshold was hit first) because more negative values of $X(T_{Dec}-\tau )-az$ support a ’lower’ decision and thus should lead to higher confidence. For perceptual decision tasks without independent manipulation of discriminability and visibility, the mean drift rate of the visibility process was previously set to the absolute mean drift of the decision process, $\mu _V=|\nu |$ (Hellmann et al., 2023, 2024). Setting the visibility drift rate to the absolute value of the decision drift rate follows the assumption that stimuli that are easier to discriminate are also perceived as more reliable, independent of their category. For example, when manipulating the stimulus-onset-asynchrony in a masked discrimination task, the time the stimulus was present on the screen may be perceived independently of the evidence about the stimulus category. Because stimuli presented longer are easier to discriminate, a longer stimulus duration increases confidence regardless of the choice. A detailed derivation of the internal confidence variable in dynaViTE and justification of the form of time penalization is provided by (Hellmann et al., 2024). DynaViTE includes simpler confidence models that were previously studied in the literature. The following special cases are implemented with their own name in **dynConfiR**.

#### Dynamical weighted evidence and visibility model (dynWEV)

The dynWEV model is a dynamic version of a previously proposed static model of confidence, the weighted evidence and visibility model (Rausch et al., 2018). DynWEV is equivalent to the dynaViTE model without considering the accumulation time penalization in the internal confidence variable, i.e., $\lambda =0$, such that

$$\begin{aligned} c_{dynWEV}=wR(X(T_{Dec}+ \tau )-az) + (1-w)V(T_{Dec}+\tau ). \end{aligned}$$

#### Two-stage dynamic signal detection theory (2DSD)

The 2DSD model does not assume parallel accumulation of visibility evidence and also has no penalization for accumulation time. In 2DSD, confidence only depends on whether the evidence accumulated in the post-decisional accumulation period supports or contradicts the choice (Pleskac, 2010). 2DSD is a special case of dynaViTE for $\lambda =0$ and $w=1$. Setting $w=1$ leads to a zero weight on the visibility process, which is thus completely ignored in the likelihood. In addition, $\lambda =0$ implies that the denominator in Eq. 1 is always 1, and accumulation time has no direct influence on confidence. The internal confidence variable has the form

$$\begin{aligned} c_{2DSD}=R(X(T_{Dec}+\tau )-az). \end{aligned}$$

#### Drift-diffusion confidence model (DDConf)

The drift-diffusion confidence model is based on the formula for optimal confidence in the DDM when drift rates are uniformly distributed (Moreno-Bote, 2010), which indicates that confidence is a monotonically decreasing function of decision time. More precisely, the internal confidence variable is defined as

$$\begin{aligned} c_{DDM}=\frac{1}{\sqrt{T_{Dec}}}. \end{aligned}$$

DDConf is mathematically equivalent to the dynaViTE model with $w=1$, $\tau =0$, and $\lambda =0.5$.

### Race models using Wiener processes

The drift-diffusion model that serves as the basis for the previously described models assumes only one accumulation process representing relative evidence for competing decision alternatives. In contrast, race models include one accumulation process for each decision alternative. Each of the processes accrues information in favor of the corresponding decision alternative. These models are theoretically applicable to decision tasks with an arbitrary number of alternatives. In the binary setting, the two accumulators may be described as a two-dimensional Gaussian process $(X_1, X_2)$ starting at (0, 0), with constant drift $(\mu _1 , \mu _2)$ and covariance matrix $$\Sigma = \begin{pmatrix} \sigma _1 ^2 & \sigma _1 \sigma _2 \rho \\ \sigma _1\sigma _2\rho & \sigma _2^2 \end{pmatrix} $$. Similar to the diffusion constant, $\sigma _1$ and $\sigma _2$ may be set to 1 as scaling factors. Each component of the process is bound from above by a time constant threshold *A* and *B*, respectively. A decision is triggered as soon as one of the accumulators hits its threshold. Decision time is thus defined by $T_{Dec}=\min \left\{ t\, |\, X_1 (t)> A \vee X_2(t)> B\right\}$ and the response *R* is 1, if $X_1(T_{Dec}) > A$ and 2, if $X_2(T_{Dec}) > B$.

In **dynConfiR**, the correlation parameter $\rho$ is restricted to either $\rho =0$, which results in the model denoted as the independent race model (IRM), or $\rho =-.5$, for which the respective model is denoted as the partially correlated race model (PCRM). The reason for restricting $\rho$ to either 0 or -.5 is that for these values, there are closed-form solutions for the first passage time densities (Moreno-Bote, 2010). Closed-form solutions allow fast, precise computation of the first-passage time distribution compared to approximation methods, which are necessary when the first-passage time density can be represented only as an infinite sum. However, these two choices of $\rho$ capture essential theoretical concepts (Teodorescu et al., 2013; Zylberberg & Barttfeld, 2012). A value of $\rho =0$ leads to an independent race of accumulators, which represents the assumption of evidence accumulation in the absence of interaction (Teodorescu et al., 2013; Zylberberg & Barttfeld, 2012). A negative correlation of the noise in the accumulation processes represents the assumption of feed-forward inhibition, which means that higher input values in one accumulator partially reduce the input in the other accumulator (Teodorescu et al., 2013; Zylberberg & Barttfeld, 2012). Note that for $\rho =-1$, the race model is equivalent to a drift-diffusion model.

One possibility to compute confidence in the context of race models is the Balance of Evidence (BoE, Vickers et al., 1985), i.e., the difference in the amount of evidence in favor of the two alternatives at the time of decision. Because the winning accumulator is always at its threshold at decision time, BoE is entirely determined by the distance of the losing accumulator to its upper threshold. For instance, if $R=1$, the internal confidence variable may thus be defined as

$$\begin{aligned} c_{BoE}=B-X_2(T_{Dec}). \end{aligned}$$

The logic behind the BoE is intuitive: the less evidence there was for the non-chosen alternative, the clearer and less ambiguous the decision, resulting in a higher degree of confidence associated with the decision. However, empirical studies have shown that confidence is also affected by decision time (Hellmann et al., 2023; Kiani et al., 2014). In addition, it has been shown that if confidence in a race model were computed optimally, it would be a function of both BoE and decision time (Moreno-Bote, 2010). For this reason, **dynConfiR** includes race models with a more general internal confidence variable in the form of a linear combination of Balance of Evidence and the inverse of decision time. Assuming again that $R=1$, confidence is computed as

$$\begin{aligned} c_{RMt} = w_X (B-X_2(T_{Dec})) + w_{RT} \frac{1}{\sqrt{T_{Dec}}} + w_{Int} \frac{B-X_2(T_{Dec})}{\sqrt{T_{Dec}}}, \end{aligned}$$

where the weights $w_X, w_{RT}, w_{Int}$ are greater than 0 and sum to 1 to form a trade-off between the possible predictor variables. Note that the fixed sum of weight parameters is not a restriction of the model because the internal confidence variable and confidence thresholds may be rescaled by the sum of weights to produce the same distribution of response time and confidence. **dynConfiR** implements race models with all combinations of assumptions about independent or correlated accumulators and an internal confidence variable that does or does not depend on decision time. The acronyms for the models used in the package are summarized in Table 2. Note that the first confidence measure $c_{BoE}$ is a special case of the more general $c_{RMt}$, if the weight parameters are set accordingly ($w_X=1, w_{RT}=w_{Int}=0$).

**Table 2:** Acronyms for the different variations of race models used in **dynConfiR**

		Internal confidence variable	
		$c_{BoE}$	$c_{RMt}$
Correlation	0	IRM	IRMt
of noise ($\rho$)	-.5	PCRM	PCRMt

### The multiple-threshold log-normal race model

The MTLNR model (Reynolds et al., 2020) forms a special case of racing accumulator models because it uses a simple ballistic accumulation instead of a noisy, Gaussian process (Brown & Heathcote, 2005). Variation in responses and response times is the result of variation in accumulation rates and boundary distances, which are both assumed to follow log-normal distributions.

In detail, MTLNR assumes log-normally distributed boundary distances for the two accumulators $(D_1, D_2)$. The distribution is described by their logarithms, $(\log D_1, \log D_2)$, which follow a normal distribution with mean $(\mu _{d1}, \mu _{d2})$ and covariance matrix $$\Sigma _d = \begin{pmatrix} \sigma _{d1} ^2 & \sigma _{d1} \sigma _{d2} \rho _d \\ \sigma _{d1}\sigma _{d2}\rho _d & \sigma _{d2}^2 \end{pmatrix} $$. Similarly, the accumulation rates $(V_1, V_2)$ follow a log-normal distribution with mean parameter $(\mu _{v1}, \mu _{v2})$ and covariance matrix $$\Sigma _v = \begin{pmatrix} \sigma _{v1} ^2 & \sigma _{v1} \sigma _{v2} \rho _v \\ \sigma _{v1}\sigma _{v2}\rho _v & \sigma _{v2}^2 \end{pmatrix} $$ for the underlying normal distribution on the log-rates.

Because of the linear ballistic nature of evidence accumulation, the boundary crossing times for each accumulator are determined by boundary distance and accumulation rate $T_i = D_i/V_i$. The first accumulator to reach its boundary determines the decision and the decision time, as in race models with Wiener processes.

The internal confidence variable in the MTLNR is defined as the logarithm of the ratio between the boundary crossing time of the losing accumulator and the boundary crossing time of the winning accumulator. For instance, if the first accumulator hits its boundary first, the internal confidence variable is computed by

$$\begin{aligned} c_{MTLNR} = \log \frac{T_2}{T_1}. \end{aligned}$$

This will always lead to values greater than 0.

Despite the formulation of an internal, continuous confidence variable, the **dynConfiR** package considers only discrete confidence judgments (see next section). In combination with the mechanism used to form discrete confidence judgments, the MTLNR implemented in **dynConfiR**  is consistent with its original formulation, which also focused on discrete confidence judgments (but see Supplementary Material section Reasoning behind the choice of the internal confidence variable; Reynolds et al., 2020).

While the MTLNR was originally formulated with a constant non-decision time component (Reynolds et al., 2020), it is implemented here with the option to include between-trial variability in the non-decision time component.

### Common mechanism in forming confidence judgments

Although models may differ in their decision architecture and the specific computation for the internal confidence variable, the mechanism for the formation of a confidence report, as implemented in the package, is the same for all models. All models are built to produce discrete confidence outcomes *C* with an arbitrary number of levels $K\ge 2$. To generate discrete confidence reports, the models assume that some internal confidence variable *c* is generated according to the formula for the respective model specified above. Confidence judgments are then determined by comparing the internal confidence variable to a set of thresholds, $\theta _{R,i}, i\in \{1,...K-1\}$, depending on the choice *R*. Formally, the reported confidence is

$$ C=\sum _{i=1}^{K-1} 1\!\!1_{(c>\theta _{R,i})}+1, $$

where $1\!\!1$ denotes the indicator function, which is one if the condition is true and zero, otherwise. This means that observers are assumed to report a confidence level of 2 on a three-point scale if the confidence variable *c* falls between $\theta _{R,1}$ and $\theta _{R,2}$.

### Non-Decision time component

All models allow for the inclusion of a non-decision time component, representing the time necessary for stimulus encoding and motor execution. This component is independent of the decision process itself but contributes to the observed response time. It is implemented as a uniformly distributed random variable $T_{ND}\sim {\text {Unif}}[t_0, t_0+s_{t0}]$ (Ratcliff & Tuerlinckx, 2002). Although the package includes between-trial variability in non-decision time, a number of studies model response times using a constant non-decision time parameter (e.g., Teodorescu et al., 2016; Tillman et al., 2020). For instance, the MTLNR was originally formulated with a constant non-decision time (Reynolds et al., 2020). Accordingly, researchers may either estimate $s_{t0}$ or set it to zero, depending on model comparison results.

The formula for the response time depends on the timing of the confidence report in the experiment at hand. For experiments in which the choice and confidence judgment were reported sequentially, the models currently implemented in the package can only account for the choice response time and do not include the confidence response time. The choice response time is assumed to be

2 $$\begin{aligned} RT=T_{Dec}+T_{ND}. \end{aligned}$$

### Application of models with post-decisional accumulation to data from simultaneous choice and confidence reports

If choice and confidence are reported simultaneously, then the response time is still defined as in Eq. 2 for models that do not assume post-decisional accumulation of evidence. For models that assume a post-decisional accumulation period, all processes are assumed to have finished at the time of the response. Thus, the observed response time is the sum of decision time, post-decisional accumulation time, and non-decision time,

3 $$\begin{aligned} RT=T_{Dec}+\tau +T_{ND}. \end{aligned}$$

However, the models with post-decisional accumulation time could also be fitted in experiments with simultaneous responses using the first definition of response time as in Eq. 2 using the same fitting functions with specific arguments (see Sect.  Fitting confidence models to experimental data).

All parameters of the different models are summarized in Table 3.

**Table 3:** List and short description of all parameters for the different models

Parameter	Description
dynaViTE	
*a*	distance between upper and lower decision boundary for decision process
*z*	relative mean starting point of decision process
$s_z$	range of uniform distribution for the relative starting point in the decision process
$\nu$	mean drift rate for decision process
*s*	diffusion constant of the decision process
$s_\nu$	variation in drift rate of the decision process
$\tau$	length of inter-rating period
$\mu _V$	mean drift rate for the visibility process
$s_V$	diffusion constant of the visibility process
$\sigma _V$	variation in drift rate of visibility process
*w*	weight on decision evidence for confidence variable
$\lambda$	exponent of accumulation time in the denominator of the confidence variable
Race Models	
*A*, *B*	thresholds for the two accumulation processes
$\mu _1, \mu _2$	drift rates for the two accumulators
$s_1, s_2$	diffusion constants for the two accumulators
$\rho$	correlation of process noise between the two accumulators
	(either 0 for IRM and IRMt or -.5 for PCRM and PCRMt)
$$ w_{X}, w_{RT},$$	weights on losing accumulator, decision time and
and $w _{Int}$	interaction for the confidence variable
MTLNR	
$\mu _{d1}, \mu _{d2}$	mean parameters for boundary distances of the two accumulators
$\mu _{v1}, \mu _{v2}$	mean parameters for accumulation rates of the two accumulators
$\sigma _{d1}, \sigma _{d2}$	variance parameters for boundary distances of the two accumulators
$\sigma _{v1}, \sigma _{v2}$	variance parameters for accumulation rates of the two accumulators
$\rho _d$	correlation between boundary distances
$\rho _v$	correlation between accumulation rates
Common parameters	
$t_0$	minimal non-decision time component
$s _{t_0}$	range of uniform distribution for non-decision time component
$\theta _{ R, k}$	set of confidence criteria, $R=-1,1,\,k=1,. ..,K-1$ for discretization into *K* steps

### Parameter identifiability in sequential sampling models

The density functions for all models are implemented with all their parameters, irrespective of scaling properties and identifiability issues. When fitted to data from a single condition, that is, when all parameters are assumed to be constant across observations, not all parameters can be identified. For instance, for DDM based models, some parameters may be scaled by a single other parameter, and therefore one of the following parameters needs to be fixed to a specific value: $s, \nu , a, s_\nu$ (Lerche et al., 2016; Ratcliff & Rouder, 1998) or alternatively, in the dynaViTE model, $\sigma _V$ or $s_V$ can be chosen as scaling parameters. Similarly, the full MTLNR model with separate mean, variance, and covariance parameters for boundary distance and accumulation rate is not identifiable (see Supplementary Material section Mathematical Details of the Multiple Threshold Log-Normal Race Model for more details). We nevertheless implemented all models with full control over all parameters. This allows the user to specify custom likelihoods with parameters that may vary across experimental conditions. If parameters vary independently across different experimental manipulations, some parameters may become identifiable. Moreover, different constraints may be put onto parameters to make the parameters identifiable, most of them implementing different assumptions about the model (see e.g., Donkin et al., 2009). What parameter constraints are best suited for the data at hand may be an important modeling decision that should be made by the user.

### Other sequential sampling models of confidence

The confidence models presented here are only a subset of previously proposed dynamic confidence models. Other models include the RTCON model (Ratcliff & Starns, 2009, 2013) or the bounded accumulation model proposed by (Kiani et al., 2014). The **dynConfiR** package is restricted to models for which closed-form solutions are available for the joint distribution of response times and confidence, or the approximations of response time distributions are well-studied concerning their precision.

## Functionalities of the package

In the following, we will first describe a prototypical workflow illustrating how the package may be used for model comparison studies. Afterward, the most essential functions implemented in **dynConfiR** are explained in detail.

**Fig. 1:**
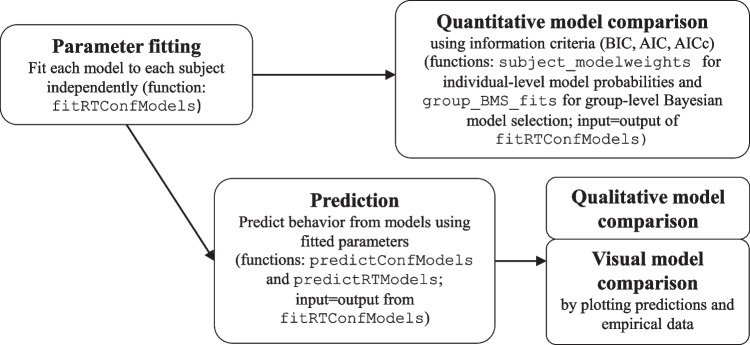
Basic workflow and functions for model comparison studies

### Installation

The package is available on CRAN and may be installed with the command:
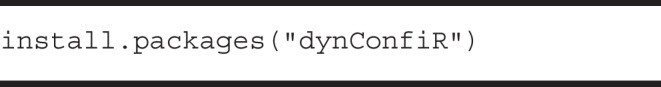


A development version of the package is available on GitHub and may be downloaded and installed using the devtools package and the command:
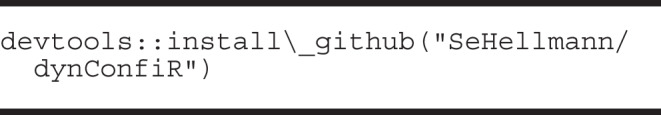


### Workflow

The **dynConfiR** package provides functions for model fitting, i.e., estimation of model parameters. The function fitRTConfModels implements the full model-fitting procedure and allows for parallelization over subjects.

The functions of **dynConfiR** are optimized for within-subjects manipulations of discriminability. Models can be fitted independently for each subject, facilitating quantitative model comparison using information criteria like AIC and BIC (Akaike, 1974; Schwarz, 1978). Firstly, since models are fitted to individual participants, computing model weights on a subject-level, which is done by the function subject_modelweights, may be used to inspect models that are prominent in the group, but also to examine heterogeneity or a group-structure with respect to the best-fitting models in the data. In many cases, a conclusion at the group level is desired. For this purpose, assuming that BIC or AIC are good approximations of model evidence, **dynConfiR** provides the function group_BMS_fit to conduct a group-level model comparison based on a Bayesian model selection approach (Rigoux et al., 2014).

Cognitive modeling studies should check whether the fitted models could reproduce the main qualitative patterns of empirical data (Palminteri et al., 2017). For this purpose, the functions predictConfModels and predictRTModels compute the predicted data distributions for given parameter sets. While predictConfModels computes the discrete decision and confidence outcomes, predictRTModels provides the density for the joint distribution of decision, response time, and confidence rating. When used with previously fitted parameters, these functions can be used to visually compare the model predictions to empirical data or to check for the reproduction of qualitative data patterns. One example of a qualitative pattern that confidence models need to explain is the relationship of mean confidence in discrimination tasks with increasing stimulus discriminability for correct and incorrect decisions, which has been referred to as a so-called folded-X or a double increase pattern (Rausch, 2019). The workflow for parameter fitting, model comparison, and prediction is summarized in Fig. 1.A step-by-step tutorial on how to use the package in a modeling study is available on the GitHub page of the package and downloadable as a R markdown document at OSF. We also illustrate the workflow in section Example of application in model comparison. In addition to classical model-comparison studies, the fitted parameters for individual subjects can be used for group comparisons and correlational analyses, for example, to examine the relationship between specific parameters and measures of metacognitive sensitivity or neurological data.

### Fitting confidence models to experimental data

In this section, we describe the fitting function in more detail, starting with which experimental data can be used, how parameters are mapped to the manipulations, and finally, the fitting procedure.

#### Experimental paradigm

The fitting functions in the package are tailored to perceptual, binary discrimination tasks with a single difficulty manipulation. This is a standard paradigm in the study of computational models of confidence (Kiani et al., 2014; Rahnev et al., 2020; Rausch et al., 2018). For other manipulations that are assumed to vary specific parameters only, the user can write their own likelihood and fitting functions using the density functions described later. For instance, manipulations of the speed-accuracy trade-off (Desender & Donner, 2021) and post-decisional accumulation period (Desender et al., 2022; Moran et al., 2015) may also be interesting when studying the formation of confidence. Still, when all model parameters are allowed to vary across conditions, the fitRTConfModels function remains applicable. In such instances, the user can specify a data column as the subject identifier, which differentiates between subject and manipulation level combinations, allowing the fitting function to fit separate sets of parameters per subject and condition. Fitting independent parameter sets for each condition can be useful for critically testing the assumption that an experimental manipulation selectively influences specific parameters (Lerche et al., 2019; Voss et al., 2004).

#### Data format

The fitting function expects the data to come in a tidy data frame, with each row representing one trial. The data frame should include the following columns (expected column names in parentheses): true stimulus identity (stimulus), binary decision response (response), categorical confidence judgment (rating), and response time (rt). As an alternative to the stimulus or response column, a column for accuracy (correct) may be provided. In addition, a column for the experimental manipulation of discriminability of the stimulus (condition) may be included, but is not necessary. Instead of renaming columns in the data frame, alternative column names may be added as arguments of the form rating = "confidence", if, for example, the column indicating the confidence rating is called confidence. A column named sbj, subject, or participant may be included to fit the models independently to individual subjects.

#### Fitted parameters

The stimulus and response categories are denoted by $S, R\in \{-1,1\}$, and task difficulty is assumed to be manipulated in *L* steps. A discrimination parameter, $d_l, l=1,\dots , L$, is fitted independently for each difficulty level. Thus, the condition column will be transformed into a factor, even when numeric values are supplied. In the confidence models, the drift rates of the different processes depend on the stimulus identity and the discriminability parameter of the difficulty level of the trial: For dynaViTE, the mean drift rate of the decision process is set to $\nu =Sd_l$, and the mean drift rate of the visibility process is set to $\mu _V=d_l$. For the race models, the drift rates are set to $(\mu _1, \mu _2) = (Sd_l, -Sd_l)$. This means that the first accumulator accumulates evidence for the first category, while the second one accumulates evidence for the category $S=-1$. For MTLNR, the mean parameters for the accumulation rates are set to $(\mu _{v1}, \mu _{v2})=(d_l, 0)$, if $S=1$ and to $(\mu _{v1}, \mu _{v2})=(0, d_l)$, if $S=-1$. Introducing an additional free parameter for the accumulation rate of the incorrect choice option would introduce a trade-off with the remaining mean parameters in the MTLNR, rendering the parameter unidentifiable (see Supplementary section Identifiability of MTLNR when fitting with fitRTConf).

All other parameters are assumed to be independent of the task difficulty manipulation and fitted for each subject. However, the diffusion constant of the decision process in dynaViTE *s* and the diffusion constants of the two processes in the race models, $\sigma _1$ and $\sigma _2$, are fixed to 1. For the DDM-based models, one parameter needs to be fixed to make the estimated parameter set identifiable. There are also other possibilities for which parameters to fix, but fixing variance parameters is a common approach in response time modeling (Lerche et al., 2016; Ratcliff & Rouder, 1998). In the race models, only one parameter for either accumulation process needs to be fixed to make the parameter set identifiable. We set both diffusion constant parameters $\sigma _{1}$ and $\sigma _{2}$ to 1, implementing the assumption that the noise in the accumulation process is identical for the two decision alternatives. This is also in accordance with previous applications of race models for confidence (Hellmann et al., 2023; Kiani et al., 2014). In the MTLNR, the variance parameters for the boundary distances $\sigma _{d1}$ and $\sigma _{d2}$ and their correlation $\rho _d$ are fixed to 0, such that only the variance parameters for the accumulation rates are fitted. Therefore, the variance components are simply labeled $s_1$ and $s_2$ for the variance parameters of the boundary hitting times of the first and second accumulator, and $\rho$ for their correlation. This is necessary to achieve identifiable parameters and is in accordance with the implementation of the MTLNR in (Reynolds et al., 2020). For more details about identifiability, we refer to the Supplementary Material.

Whether choice and confidence were reported simultaneously or sequentially is determined by the simult_conf argument, which should be set to TRUE if the reports were given simultaneously and FALSE otherwise.

The number of confidence thresholds $\theta _{ R, k}, k=1,\dots , K-1$ separating the internal confidence variable into discrete steps depends on the number of possible levels for the discrete confidence rating *K*. The confidence thresholds can vary between choice responses by default, leading to $2(K-1)$ fitted confidence threshold parameters. We recommend specifying the nRatings argument to provide the number of confidence levels because not every subject might have used the full range of the scale. Alternatively, the ratings column can be provided as a factor with factor levels representing the possible rating outcomes. If not all confidence levels were used, the number of fitted parameters is reduced internally because the maximum likelihood is attained by some thresholds being identical in this case. If the lowest (or highest) confidence level was not used, then the likelihood is maximized by setting the lowest confidence threshold to minus infinity (or the highest threshold to infinity). If an intermediate confidence category is not used, the likelihood is maximized when the two confidence thresholds are identical. Therefore, the concerned confidence thresholds do not need to be optimized numerically by the optimization procedure but may be set afterward to speed up the optimization. The nRatings argument is required to correctly format the output parameters and report the right number of fitted parameters.

To sum up, the total number of parameters depends on the number of steps in the manipulation *L* and the number of levels for the discrete confidence rating, *K*. This means that for dynaViTE, there are $11+L+2(K-1)$ parameters. In the race models with a time-dependent internal confidence variable, there are $6+L+2(K-1)$ parameters. Two of the three weight parameters must be fitted, while the third is determined by the condition that the sum of the weights equals 1. In addition, the correlation parameter $\rho$ is not estimated but fixed at -0.5 for PCRMt and 0 for IRMt. For MTLNR, the number of fitted parameters is equal to $7+L+2(K-1)$. For special cases like 2DSD or race models with a time-independent internal confidence variable, the number of parameters is reduced by the number of fixed parameters. In addition, if the confidence thresholds are assumed to be symmetric for the two response options (by setting fixed=list(sym_thetas=TRUE)), the number of parameters is reduced by $K-1$.

Which models should be fitted is specified by the models argument. The function fitRTConfModels allows for all models presented in the section Sequential sampling confidence models: dynaViTE, dynWEV, 2DSD, DDConf, IRMt, PCRMt, IRM, and PCRM. In addition, the user may fix individual parameters by providing the argument fixed in the form of a list. For instance, researcher may want to assume an unbiased observer by setting $z=0.5$ for the drift-diffusion-based models and $A=B$ for the race models. Moreover, specifying sym_thetas=TRUE in the list leads to symmetric confidence thresholds for the two choice possibilities, i.e., $\theta _{1, k}=\theta _{-1, k} \forall k = 1,\dots , K-1$.

#### Fitting procedure

The function fitRTConfModels fits the models specified in the models argument to each individual subject in the data set using maximum likelihood estimation, i.e., by minimizing the negative log-likelihood of model parameters. The likelihood is computed under the assumption of independent observations, which means that for trials $i=1,...,N$; the vectors for presented stimulus identity *S* and task difficulty *D*; and the vectors for observed outcomes response time *RT*, confidence rating *C*, and response *R*, the negative log-likelihood of a set of parameters $\vartheta$ is computed as

$$ \mathcal {L}(RT, C, R | \vartheta , S, D) = -\sum _{i=1}^{N}\log \mathbb {P}(RT_i, C_i, R_i | \vartheta , S_i, D_i). $$

The optimization procedure starts with a grid search, in which the likelihood is computed for a broad range of possible parameter combinations. The best-performing parameter sets identified in the initial grid search are used as starting values for the optimization algorithm. The optimization algorithm is restarted several times, using the previous run’s output as the starting point for the next run, to help it avoid local minima. The number of initial values and restarts for the optimization procedure can be set by the user using the opts argument. The functions allow parallelization across both subject-model combinations and within a single fitting procedure over the starting values for optimization.

The output is a data frame with one row for each combination of subject and fitted model. The columns of the output data frame are the fitted model parameters together with additional information, like the number of trials (N), fixed parameters (fixed), and the following performance measures: the final negative log-likelihood and model selection criteria AIC, AICc, and BIC.

On the one hand, the maximum-likelihood fitting procedure implemented in fitRTConfModels is an efficient way for estimating parameters using all the available information in the data without aggregating to quantiles (Lerche et al., 2017; Voss & Nagler, 2013). On the other hand, the maximum likelihood method is known to be influenced by contaminant response times, which are not generated by a DDM (Lerche et al., 2017; Ratcliff & Tuerlinckx, 2002). Therefore, it is recommended to apply a filter on trials at the level of the individual subject, e.g., by removing trials with response times that are either below a certain threshold (e.g., 300 ms) or which deviate significantly from the mean or median of the response time distribution (e.g., response times, which exceed the mean plus two standard deviations; Hellmann et al., 2023; Pleskac, 2010). There are different strategies for removing contaminants in the data. Some studies use hard cut-offs (Lerche et al., 2017; Ratcliff et al., 2004; Van Den Berg et al., 2016), others use exclusion criteria based on the interquartile range (Lerche et al., 2019; Voss & Nagler, 2013) or alternatively, a mixture of hard cut-off for the fast responses and a distribution-dependent cut-off for slow responses (Hellmann et al., 2023, 2024; Lerche et al., 2017; Moran et al., 2015; Pleskac, 2010). However, it is hard to suggest general guidelines for exclusion criteria that suit all experiments. For new experiments, we recommend using pilot data to infer suitable exclusion criteria because different experimental paradigms produce different response time distributions. In light of ongoing replication issues in psychology (Röseler et al., 2024), we recommend pre-registering exclusion criteria and checking whether results are robust concerning the specific choice of exclusion criteria (Wagenmakers et al., 2012).

#### Predicting confidence and response time distributions

Empirical data are often visually compared with model predic-tions to detect qualitative mismatches. For this purpose, **dynConfiR** includes the functions predictConfModels (for the discrete decision and confidence distribution) and predictRTModels (for the joint distribution of response time, decision, and confidence). These take data frames with parameters as input. Notably, the output of the fitting procedure can be directly inserted into the prediction functions. predictConfModels returns a data frame with columns for stimulus identity, response, and confidence judgments, and a column indicating the probability of an outcome. predictRTModels has an additional column for the response time, spanned equidistantly for a user-provided interval. If the input has more than one row, columns for subject ID and model are required, and the output will be accordingly structured by binding the data frame outputs for each subject and model combination one below the other. Similarly to the fitting function, participant, subject, and sbj column names are accepted as identifiers.

#### Quantitative model comparison using information criteria

Besides the qualitative and visual inspection of model fits, a quantitative model comparison and model selection is often conducted to compare models when qualitative data patterns are not diagnostic (Farrell & Lewandowsky, 2018). The information criteria BIC, AIC, and AICc, are used to approximate the negative log-model evidence, such that Bayesian approaches for model comparison may be used. On a subject level, this information criterion can be directly transformed into model weights to assess the prevalence of certain models, but also heterogeneity within the sample. This is done by the function subject_modelweights, which takes a data frame with columns for model names, subject identifier (one of participant, subject, or sbj), and the information criterion that should be used as input. The second argument measure gives the name of the column with the information criterion, and is "BIC" by default. Therefore, the output of a fitRTConfModels call may be directly used as an argument for the function subject_modelweights.

Similar to the subject-level model comparison, the function group_BMS_fits takes the result of model fitting but performs a group-level Bayesian model selection based on a random effects model of model prevalence across subjects (Rigoux et al., 2014; Stephan et al., 2009). The random effects model assumes a Dirichlet distribution for model probabilities in the population, for which the $\alpha$ parameter is estimated based on a variational Bayesian approach and algorithm described in (Stephan et al., 2009). The estimated parameter may be used to calculate models’ exceedance probabilities. The exceedance probability of a model is defined as the probability that the model has a higher probability compared to all other models given the Dirichlet distribution. The function also provides a scaled version of the exceedance probabilities, the protected exceedance probabilities (PEP). PEP controls for the Bayesian omnibus risk (BOR), which quantifies the risk of assuming the random effects model in contrast to a null-model, in which all models have always the same prior probability, i.e., the limit of a Dirichlet model with an $\alpha$ parameter with equal components that approach infinity. The estimation of BOR is based on a variational approach, and the implementation is based on the VBA toolbox for Matlab (Daunizeau & Adam, 2014).

In addition to the protected exceedance probability and exceedance probability, the function group_BMS_fits calculates the model probabilities based on a fixed effect model that assumes that there is a single-best model in the population. The latter is equivalent to calculating model weights based on the sum of BIC values across all participants. By default, model comparison is performed using the BIC. Using the second argument, measure, one could base the comparison also on the AIC or AICc in both functions. We decided to use the BIC as the default, as it is the most conservative criterion for the number of data points typically used in studies modeling response times.

### Other functions

#### Probability density functions


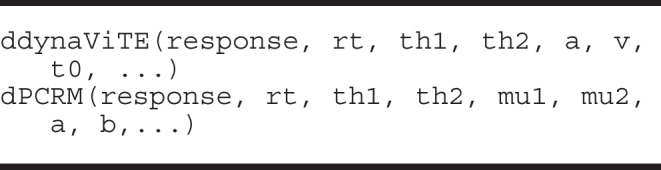


The implemented confidence and response-time distributions serve as the basis for model fitting and predictions. The distributions are implemented as probability densities in C++ and accessed in R using Rcpp. The usage of the different density functions is very similar. The first arguments represent the outcome variables: response time (*RT*), the binary choice (*R*), and the interval for the internal confidence variable ($\theta _1$ and $\theta _2$). The density functions return the probability $\mathbb {P}_{\text {model}}(RT, R, c_{model}\in [\theta _1, \theta _2] | \vartheta )$. The model parameters are passed as additional, individual parameters. Note that $\theta _1$ and $\theta _2$ are also parameters usually estimated during model fitting. The densities for drift-diffusion-based models are approximated using the truncated series for the density of the drift-diffusion model (see Voss et al., 2004; Navarro & Fuss, 2009). The densities for the race models are implemented according to the formulas in (Moreno-Bote, 2010), which are derived using the methods of images for the stochastic differential equation. The integration over the distributions of starting points and non-decision time components is performed numerically using a rectangular approximation with equidistant steps (see the Precision analysis section). The density functions may be used for theoretical calculations and for implementing other model-fitting algorithms instead of the maximum-likelihood estimation procedure included in **dynConfiR**.

#### Log-likelihood functions


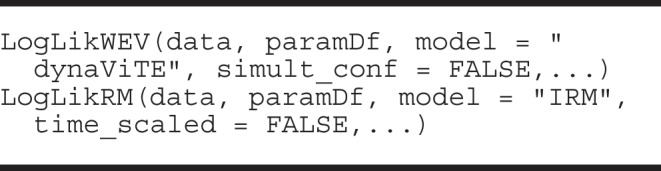


There are also functions for calculating the log-likelihood of a data set given some parameters for each model. The two main arguments are data, a data frame of the empirical data with the stimulus, response, response time and confidence, and paramDf, a data frame with one row and columns for the required parameters of the chosen model. The log-likelihood function is included in **dynConfiR** mainly to allow for the investigation of the impact of experimental manipulations on specific parameters or other relationships between stimulus discriminability and mean drift rate. For example, previous studies assumed a power function for the relationship between physical stimulus intensity and internal signal strength (Ratcliff et al., 2018; Teodorescu et al., 2016) instead of fitting discriminability parameters for each level of the experimental manipulation. The likelihood functions are wrappers of the density functions that can be used easily in custom-built cost functions for optimization.

#### Simulation functions


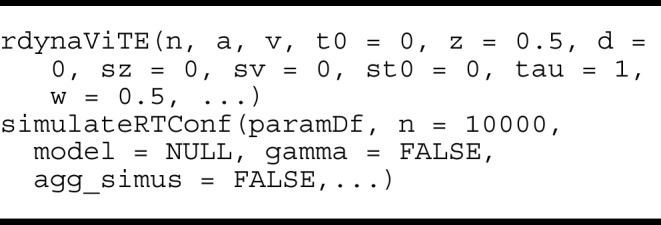


The package includes low-level and high-level simulation functions. Because simulation is based on a discretization of the stochastic differential equation, there is an argument delta determining the step-size of the discretization and an argument maxrt, which determines the maximal simulated decision time. The simulation of a single trial is stopped when the stopping criterion has not been met and the maximum decision time has been exceeded. When the simulation is stopped without a choice, a response of 0 is returned. First, the low-level simulation functions are similar to the simulation functions of other probability distributions in **R**, e.g., rdynaViTE and rPCRMt, with arguments for each parameter, most of them having default values.

A high-level function for simulating data with fitted parameters is also available. The function simulateRTConf takes a data frame with one row and columns for the required parameters. The high-level function simulates n trials per stimulus identity (which stimulus identity is used for the simulation may be changed with the stimulus argument) and difficulty condition. The number of difficulty levels is determined by the number of drift rates in the paramDf argument. To simplify the application to several parameter sets and models, the model argument can be given as a column in the paramDf argument. In addition, simulateRTConf offers the possibility to aggregate the output over response times, i.e., reporting only the discrete outcomes of choice and confidence. Finally, when gamma=TRUE, the function computes Kruskal’s gamma (Nelson, 1984) between confidence and several other relevant variables, e.g., between confidence and accuracy for different levels of stimulus discriminability. If gamma=TRUE is used, the output is a list with two components: simus for the data frame with the actual simulated data and gamma with several data frames for different gamma correlations.

## Example of application in model comparison

Now, we present a complete example of an analysis including a model comparison. The data set for this demonstration was generously published by Law & Lee (Ng et al., 2021) and was downloaded from the confidence database (Rahnev et al., 2020). The data set is available at https://osf.io/vgr27.

### Experimental method

The study was initially conducted to investigate serial dependence in confidence judgments using random-dot kinematograms. Sixteen participants reported their perceived motion direction, which was either leftwards or rightwards, simultaneously with their confidence using the keyboard. Confidence was reported on a four-point scale.

Task difficulty was manipulated by varying motion coherence. In a 240-trial calibration phase, coherence values for target accuracy levels of .52, .65, and .78 were determined using a staircase technique. The resulting coherence values were then used in the experimental phase for coherence levels 1, 3, and 5, respectively, while the coherence values for the second and fourth levels of the manipulation were determined by averaging the values for the first and third level and the third and fifth level, respectively. The experimental trials consisted of 20 blocks with 60 trials each. Because of the primary aim of the study, trials with medium difficulty, i.e., a coherence level of 3, were always preceded by either one or two trials with either high (level 1) or low (level 5) difficulty. This trial-by-trial dependency will be ignored in the following analysis.

Participants did not receive trial-by-trial feedback but instead received feedback at the end of each block about both their overall accuracy and their accuracy in the previous block.

### Data

After downloading the data from the confidence database, we selected the relevant columns, converted their names to lowercase, and removed the calibration trials from the data set. We then renamed the columns for the subject ID to participant (note that the functions accept the column names subject and sbj as well) and response times to rt. As confidence was measured on a four-point scale, we did not have to bin the rating response. The resulting data set has the following form. 
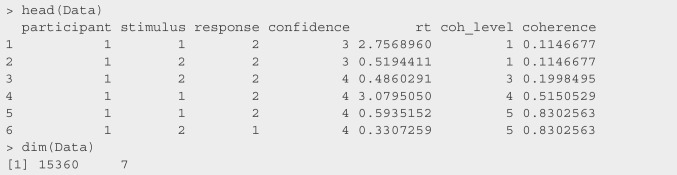


### Data preprocessing

A typical step before fitting sequential sampling models is to remove potential outliers from the empirical response-time distribution. Filtering individual data by response times is recommended because the maximum likelihood method, which is used by **dynConfiR**, is known to be specifically influenced by outliers, and there is no implementation of lapses in the current version of the package.

We removed responses that were faster than the median minus one standard deviation and slower than the mean plus 4 standard deviations for each participant, resulting in the removal of 1.7% of all trials.

In the second step, we removed one participant because they did not perform above chance (0.53 compared against 0.5), which delivered no evidence of being above chance in a Bayesian proportion test against chance level accuracy with a prior scale conducted via the proportionsBF function from the BayesFactor package (Morey et al., 2024) with a prior scale parameter of 0.5.

### Analyses

#### Model fitting

We wanted to conduct a model comparison on the data, comparing a broad range of possible models. We considered models previously compared on similar datasets from visual discrimination experiments: dynaViTE, dynWEV, 2DSD, PCRMt, and IRMt (Hellmann et al., 2023, 2024). Note that although we did not explicitly fit all models, simpler models are special cases of the models fitted in this section. For instance, race models with time-independent internal confidence variables, IRM and PCRM, are special cases of IRMt and PCRMt, respectively. In addition, we fitted the MTLNR model, which was not previously compared to the above models on empirical data. The first step was to fit the model parameters for each participant, which was achieved with the following command:
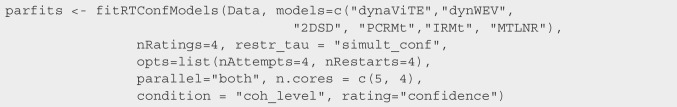


The function fitRTConfModels splits the data for the participant column and fits each of the models given in the respective models argument. Providing nRatings=4 ensured that three confidence thresholds are fitted for participants who did not use the full range of the confidence scale. The argument restr_tau="simult_conf" indicates that choice and confidence responses were reported simultaneously.

**Fig. 2:**
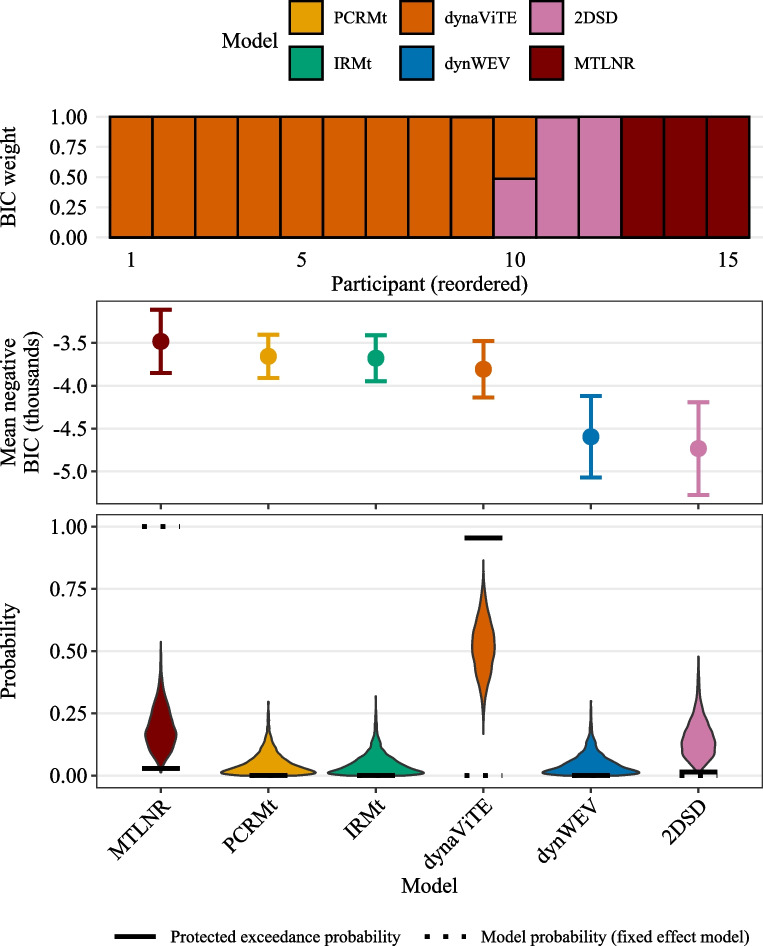
*Upper panel*: BIC weights across participants (reordered). *Middle panel*: Negative mean BIC values. *Error bars* represent within-subject standard errors. *Lower panel*: Results from a Bayesian model selection analysis. *Violin plots* show simulated model probabilities from a Dirichlet distribution fitted to BIC values according to a random effects model. *Solid bars* indicate the corresponding protected exceedance probability, *dotted bars* indicate the model probabilities resulting from a fixed effect model

The argument opts is optional and allows for adaptations of the fitting procedure. We used only the four best parameter sets (default: 5) from the initial grid search as starting values for the optimization routine and started the optimization algorithm with a broad trust region four times (default: 5).

With the combination of parallel=TRUE and n.cores =c(5,4), we distributed the fitting procedure across different CPUs, fitting five participants in parallel with four cores per participant. Therefore, for each participant, the four optimization routines with the different initial parameter settings were run in parallel.

Finally, it was necessary to include column names that deviate from the default ones, which was achieved with the last two arguments.

#### Quantitative model comparison

With a few lines of code, the first central part of the analysis was achieved. With the outputs, a quantitative model comparison may be conducted using the information criteria available in parfits. Information criteria for models fit to participants independently can be used in different ways for model comparison. Model comparison could be performed at the participant level by calculating BIC weights independently for each participant. This approach may already indicate dominant individual models, but it can also represent heterogeneity across participants or groups of participants. Still, it is sometimes desirable to investigate model performance at the group level. The results for both analysis levels are easily obtained by using the functions subject_modelweights respectively group_BMS_fits with parfits as input.
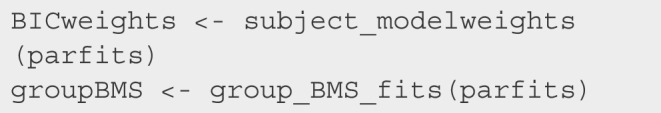


In the following, we visualize only the results based on the BIC. The **dynConfiR** package also includes AIC and AICc in the output, which are not visualized here because the results are almost identical. On a participant-level, the dynaViTE model provided the best account for nine out of 15 participants, while the MTLNR model provided the best account for three participants (Fig.2, upper panel). Visualizing the average BIC values over the sample, we see that the MTLNR and the race models achieved the lowest average BIC (Fig. 2, middle panel). Among the DDM-based models, dynaViTE performed best. Despite the close match between the two race models, the results of the group-level model selection according to a fixed effect model clearly favor the anti-correlated race model (dotted lines in the lower panel of Fig. 2). In contrast, the random effects model decisively favors the dynaViTE model as indicated by protected exceedance probability ($pep_{dynaViTE}=.99$, Figure 2).

Supplementary Fig. 2 demonstrates that the average differences and thus the fixed effect model probabilities are dominated by two participants, who showed an extreme difference in BICs between race models and DDM-based models. Inspecting the Bayesian omnibus risk for the fixed-effect model relative to the null model, which is indistinguishable from 1, indicates that the fixed-effect model is generally a poor fit to the data due to heterogeneity. In contrast, the random effects model yields a BOR of .0016, clearly indicating a better account of the data.

#### Prediction and visual model fit

The output of the fitting function may be passed directly to the prediction functions, which also use parallelization over participants. The prediction functions automatically split the first data frame argument by participant and model columns, and select the appropriate prediction function for each row’s model.
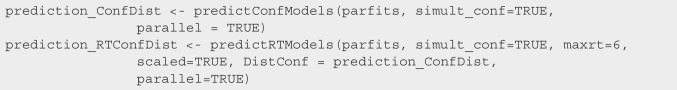

**Fig. 3:**
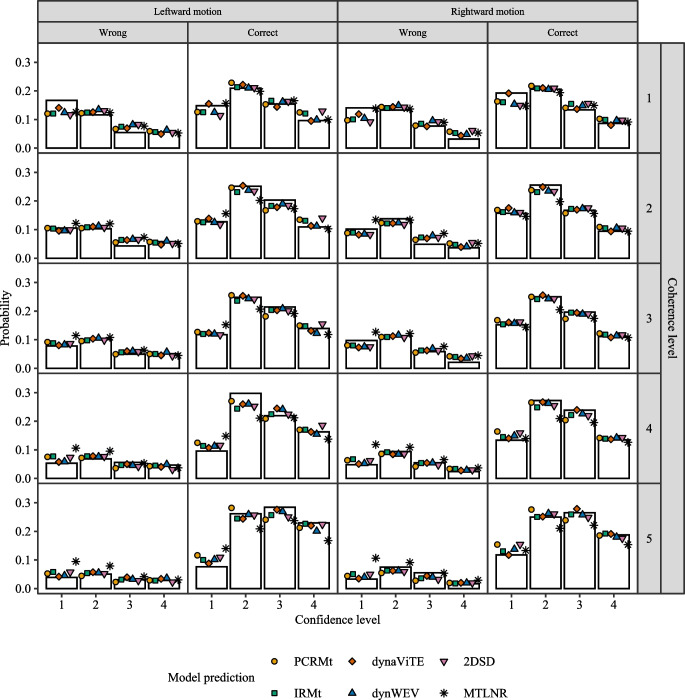
Observed (*bars*) and predicted (*points*) response distribution for the different models (*shape and color of points*) across stimulus identity (*columns*, high level) and levels of stimulus discriminability (*rows*). Probabilities within each row and stimulus identity column add to 1 for each group of data shown, i.e., height represents the conditional probability of a given accuracy and confidence rating given the stimulus discriminability and identity

**Fig. 4:**
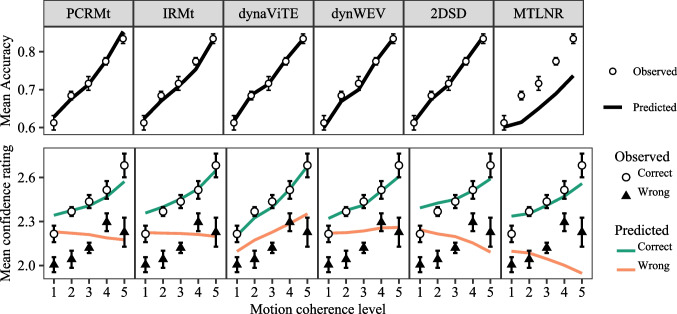
Accuracy (*top row*) and mean confidence rating (*bottom row*) for empirical data (*points* and *triangles*) and model predictions (*lines*). *Error bars* represent within-subject standard errors

Furthermore, visualizations may be generated with the predictions to compare model fits with empirical distributions. The output of the function predictConfModels has the following form (note that only the first digits are printed for readability):
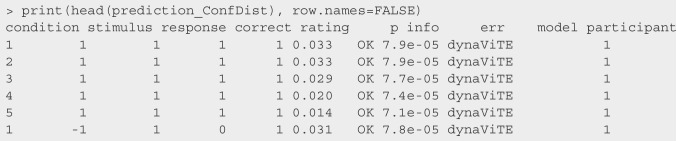


In the above data frame, p represents the probability of a confidence rating and response given the stimulus identity and discriminability condition in the respective row. The columns info and err reproduce the output of the call to integrate used to compute the probabilities. We can compare the predicted distribution to the observed data distribution (see Fig. 3), which can be used to assess the overall precision of the model fit. It seems that all models fitted the overall data pattern well. The strongest deviation is the overestimation of low-confidence responses for easy conditions in the MTLNR. In addition, there is generally a slight overestimation of low confidence for correct leftward responses in easy conditions, particularly in the race models (IRMt and PCRMt). In addition, for difficult stimuli, the probability of very low confidence was underestimated, while it was overestimated for high confidence. Interestingly, for all other conditions, there was a tendency to underestimate confidence in leftward motion choices (second and third columns) but to overestimate confidence in rightward motion choices (first and fourth columns). Participants seem to have shown a motion-dependent confidence bias leading to higher confidence ratings for correct leftwards motion responses compared to rightwards motion responses, which all the models, but particularly the three race-based models, were not able to capture very accurately (Supplementary Fig. 3).

Using the full response distribution, it is possible to aggregate on different levels to examine specific data patterns. One possibility is to visualize the increase in accuracy with easier decisions (Fig. 4, top row), for which the MTLNR shows a strong underestimation of accuracy in medium to high discriminability trials. Researchers might also be interested in the relationship between confidence and stimulus discriminability for correct and incorrect decisions (Fig. 4, bottom row). In the present example, we see increasing mean confidence with higher stimulus discriminability for both correct and incorrect decisions, which is referred to as a double-increase pattern (Rausch, 2019). Many computational models of confidence are not able to produce such a pattern but can only account for a negative relationship between confidence and discriminability in incorrect decisions, resulting in the folded-X pattern (Hellmann et al., 2023; Rausch et al., 2020, 2018). One example of a model that only accounts for a folded-X pattern is the 2DSD model, which also showed this pattern in the present example. Although the PCRMt and IRMt are in principle able to account for a double-increase pattern, they showed a flat curve for incorrect responses, indicating constant confidence across difficulty levels for incorrect choices. Finally, the MTLNR also produces a folded-X pattern. The dynWEV model underestimated the steepness of the increase in confidence for incorrect decisions. The dynaViTE model showed the most pronounced double-increase pattern, but overall overestimated confidence in incorrect decisions.

**Fig. 5:**
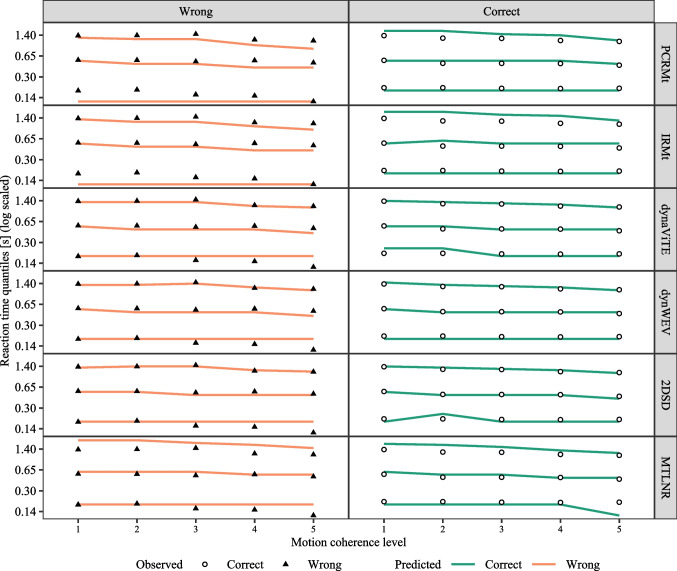
Response time quantiles for observed (*points*) and predicted (*lines*) response time distributions across correct and incorrect decisions (*columns*) and levels of stimulus discriminability (*x-axis*). Probabilities for quantiles: .1, .5, .9

**Fig. 6:**
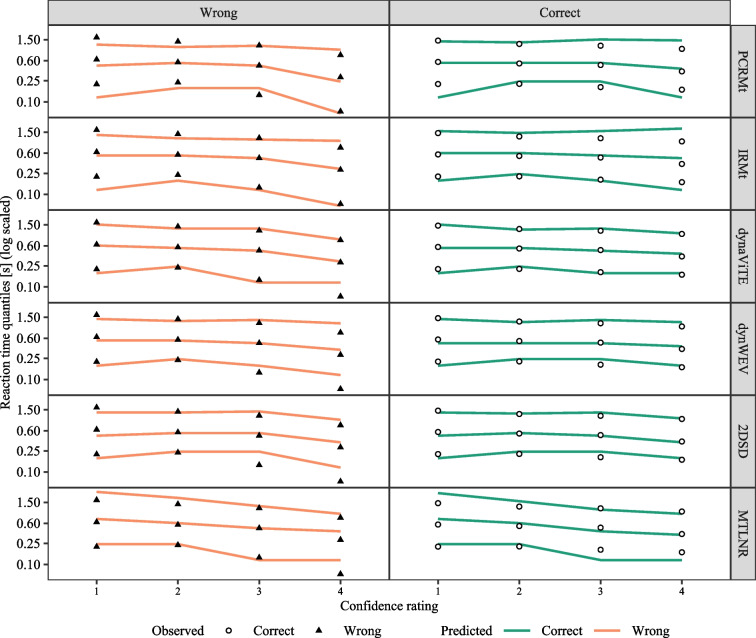
Response time quantiles for observed (*points*) and predicted (*lines*) response time distributions across correct and incorrect decisions (*columns*) and confidence ratings (*x-axis*). Probabilities for quantiles: .1, .5, .9

To compute the predicted response time distributions for the fitted parameters, **dynConfiR** offers the predictRTMo dels function, which, similarly to the predictConfMo dels, computes the joint distribution of choice, confidence rating, and response time for each level of stimulus identity and discriminability. The dens column represents the defective distribution of response times, i.e., the integral of the density for each confidence rating and response is not 1 but equals the probability of the respective choice and confidence report. With the argument scaled=TRUE, the correctly scaled densities (densscaled) are computed by dividing the defective density values by the probability of the respective discrete response. For this purpose, an additional argument (DistConf) may be provided by passing the output of predictConfModels to prevent the repeated computations of the discrete distributions. Note that the DistConf argument must have the same participants, models, and response and stimulus coding used for the models as the first argument. The resulting data frame has the following form:
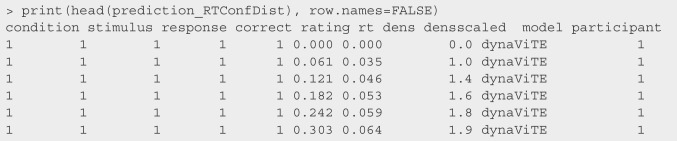


In the decision-making literature, response time distributions are commonly visualized using quantiles (Figures 6 and 5, Ratcliff et al., 2004). For convenience, the package includes the function PDFtoQuantiles, which computes quantiles from a vector of probability density values. It also allows data frames with multiple columns that can be used as groups (e.g., for computing quantiles across different experimental conditions or participants). The relationship between task difficulty and response times was rather weak in the present example, illustrated by the flat quantile curves in Fig. 5. Response times decreased slightly for easier stimuli, as captured by all models. Here we see that the most notable deviation from the data is produced by the MTLNR in the correct choices for the easiest condition, for which the MTLNR underestimates the lower tail of the distribution. Concerning the relationship of confidence with response times, there was a weak negative relationship, which was more pronounced in incorrect choices (Fig. 6). This pattern was again well captured by all models. The most pronounced deviations are visible in incorrect choices, for which the race models slightly overestimated the decrease in response times in the upper quantiles, and the 2DSD model did not reproduce the speed up for the lowest quantile in high confidence.

**Fig. 7:**
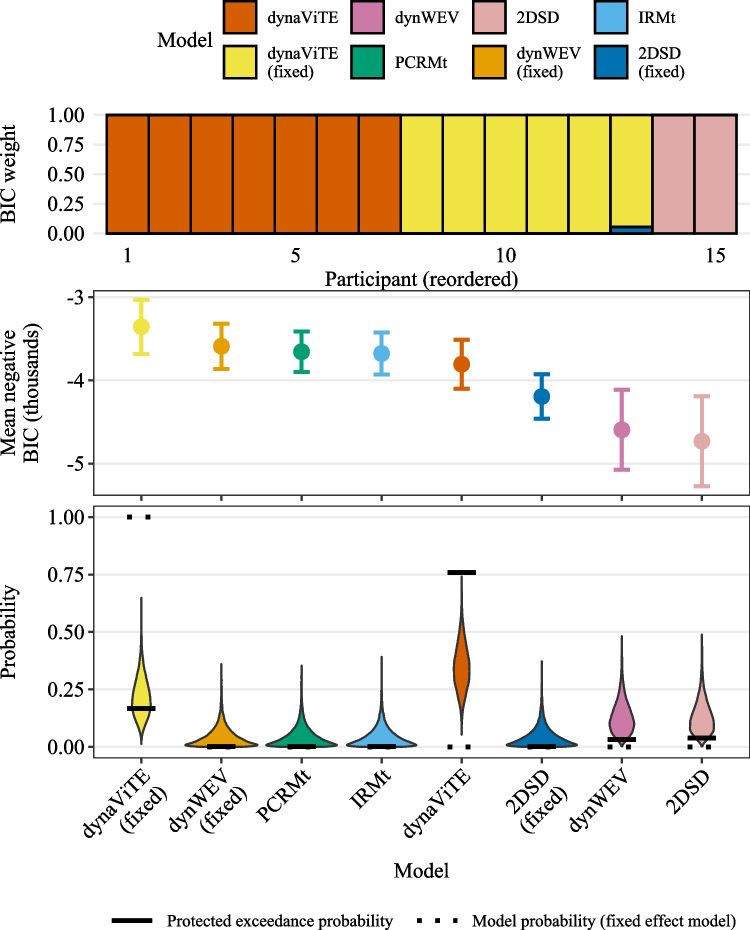
*Upper panel*: BIC weights across participants (reordered). *Middle panel*: Negative mean BIC values. *Error bars* represent within-subject standard errors. *Lower panel*: Results from a Bayesian model selection analysis. *Violin plots* show simulated model probabilities from a Dirichlet distribution fitted to BIC values according to a random effects model. *Solid bars* indicate the corresponding protected exceedance probability, *dotted bars* indicate the model probabilities resulting from a fixed effect model

#### Exploratory analysis: Fixing model parameters

The drift-diffusion-based models, i.e., DDConf, 2DSD, dynWEV, and dynaViTE, include all between-trial variability parameters. It is easy to fit these models additionally without allowing for between-trial variability in the starting point and non-decisional time by setting the respective parameters to 0 in the fitting routine. The following code demonstrates the fixed argument. We additionally fixed the diffusion constant in the visibility process for dynWEV and dynaViTE to 1, equal to the diffusion constant in the decision process. Note that fixing any parameter only affects the models that include those parameters. After the model-fitting procedure, we renamed the models for the more restricted versions and combined all parameter fits into a single data frame.
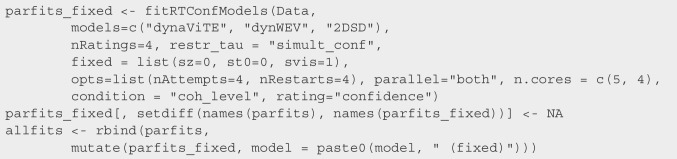


The quantitative comparisons show that the restricted dynaViTE model performed best on an average level as well as for six individual participants, while the full dynaViTE model still fitted best for seven participants. Although the restricted dynWEV and the PCRMt model still had the second and third best average BICs, no participant was best fit by either model. This illustrates again that quantitative model comparisons can lead to different results when performed on a group level compared to the individual participant level (Fig. 7). Over the extended set of models, the fully flexible dynaViTE still achieved a PEP of .75, the restricted version of the dynaViTE a PEP of .17, and the full 2DSD of .03. Concerning the visual model fit, the restricted models did not deviate strongly from the more complex models (Supplementary Figs. 4-6).
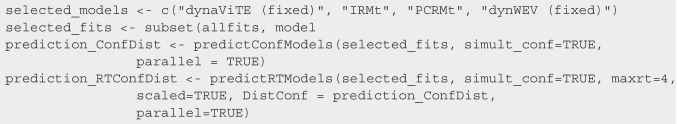


## Parameter recovery analysis

In order to compare fitted model parameters between groups or within subjects across different experimental conditions, it is necessary that the estimation of model parameters is robust. To assess the robustness of the model fitting procedure we conducted a parameter recovery analysis using artificial, data sets for the four most general models: dynaViTE, PCRMt, IRMt, and MTLNR, which include other models implemented in **dynConfiR** as special cases.

### Method

To assess parameter recovery, we generated artificial data sets using known parameter values and then refitted each model to the simulated data. This procedure allowed us to evaluate how well the recovered parameters matched the generating ones. We assumed five confidence levels ($K=5$) and five stimulus discriminability levels ($L=5$), resulting in 26 fitted parameters for dynaViTE, 21 for IRMt and PCRMt, and 22 for MTLRN. Generating parameter sets were drawn from previous empirical model fits (Hellmann et al., 2023, 2024; Ng et al., 2021; Orchard et al., 2022; Shekhar & Rahnev, 2021). Confidence thresholds were not taken directly from these fits but were instead computed from quantiles of the simulated confidence variable, ensuring all confidence levels were represented.

Parameter recovery was evaluated across different data set sizes (50–500 trials per condition) using the concordance correlation coefficient (CCC; Lin, 1989) as a measure of agreement between generating and recovered parameters. Full methodological details and preprocessing steps are provided in the Supplementary Material.

#### Results

Figure 8 illustrates the parameter recovery performance as measured by the concordance correlation coefficients across model parameters (also see Supplementary Figs. 7-22 for more detailed results). The results indicate that the decision-related parameters are generally more robust to recover for all models but the MTLNR.

In dynaViTE, the only choice-specific parameter that is hard to recover is the between-trial variability in the starting point (*sz*). Concerning the confidence-related parameters in dynaViTE, only the diffusion constant in the visibility accumulation ($s_{Vis}$) has a relatively low CCC, which requires at least 200 trials per condition and stimulus identity (2000 trials in total) to achieve a CCC of .57. A possible solution to improve the robustness of parameter fitting might be to fix $s_{Vis}$ to 1, similar to the diffusion constant in the decision process.

For race models, the confidence thresholds show a slightly lower recovery rate, although the general recovery is relatively good, even with fewer trials. The recovery of the confidence thresholds may also be increased by assuming a parametric relationship between them instead of fitting each threshold independently and only restricting them to be monotonic.

In MTLNR, the decision parameters show, in general, slightly worse but still satisfying recovery. Importantly, the recovery does not strongly improve with the number of trials.

**Fig. 8:**
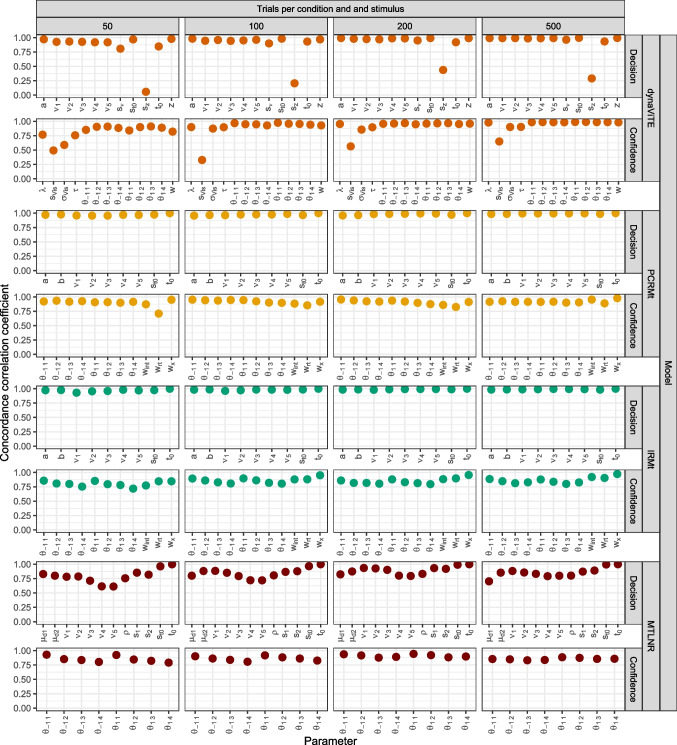
Concordance correlation coefficients (Lin, 1989) between the true and recovered parameters from the parameter recovery across the number of trials per condition and stimulus identity (*columns*) and generative model (*rows*)

**Fig. 9:**
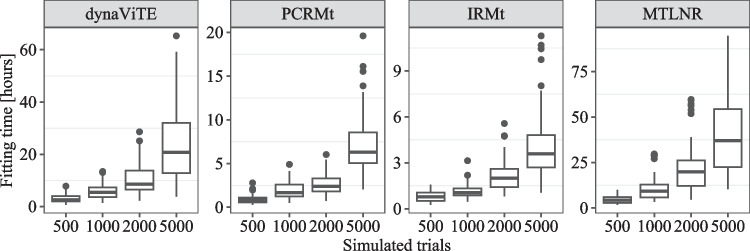
Fitting times for the simulated data sets in the parameter recovery

Figure 9 shows the time it took to fit the parameters to each data set. This illustrates that, without parallelization, fitting times may exceed several hours for large data sets, which limits the application of the package in such situations to machines with sufficient computing power. In addition, the MTLNR and dynaViTE models take considerably longer compared to PCRMt and IRMt with fitting times exceeding 48 h for big data sets.

**Fig. 10:**
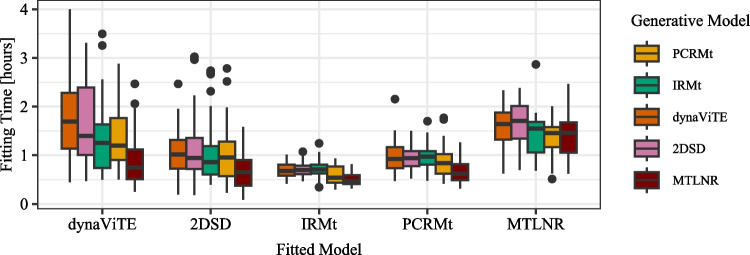
Fitting times for the simulated data sets in the model recovery

## Model recovery analysis

Besides the importance of robustly estimating model parameters for each individual model, it is important to identify the true data-generating model among a set of candidate models, given that some model is reasonably close to the data-generating process.

We assessed model recovery in a manner similar to parameter recovery, simulating data for 4 of the implemented models: 2DSD, dynaViTE, IRMt, and PCRMt. We then fitted each of these four models to the artificial datasets. Based on the fits, we identified the best-fitting model in terms of PEP for the entire simulated sample using the implemented group_BMS_fit function. In addition, we classified models based on the minimum BIC, AIC, and AICc for each simulation and computed classification precision.

### Method

For the simulation of artificial data, we used the same method as in the parameter recovery analysis, sampling from parameter sets obtained by previous model fits to empirical data.

We sampled 50 parameter sets for each of the following models: 2DSD, dynaViTE, IRMt, PCRMt, and MTLNR. For each parameter set, we simulated artificial data with 100 trials per combination of stimulus identity and discriminability, resulting in 1000 trials per individual.

We then fitted each of the five models used for data generation to each of the simulated data sets. We fitted the models using the initial grid search, the four most promising parameter sets for two iterative calls to the optimization algorithm each. We reduced the number of optimization routines to speed up the fitting. The results show, however, that model recovery is still very good.

#### Results

Figure 10 shows the time needed to fit each data set. The race models are generally fitted faster, with an expected time of 1 h for the PCRMt and slightly less for IRMt. For 2DSD, most of the cases also take 1 h, however values up to 2 h occasionally occur. The longest fitting times are observed for dynaViTE – the most complex model – with up to 4 h, while most cases fall below 2 h. MTLNR shows the highest median fitting time with around 1.5 h, despite the relatively small number of parameters. This is probably due to the high numbers of steps in the numerical integration necessary to achieve a sufficient precision in the likelihood (see Sect. “Precision analysis”).

**Fig. 11:**
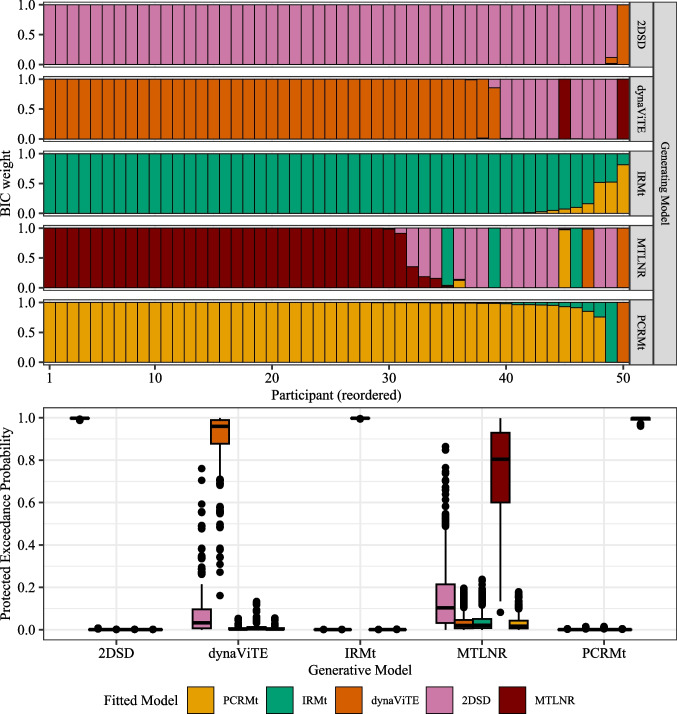
*Upper panel*: Individual model weights for each generative model (*rows*) across simulated participants. *Lower panel*: Bootstrapped protected exceedance probabilities (PEP)

**Fig. 12:**
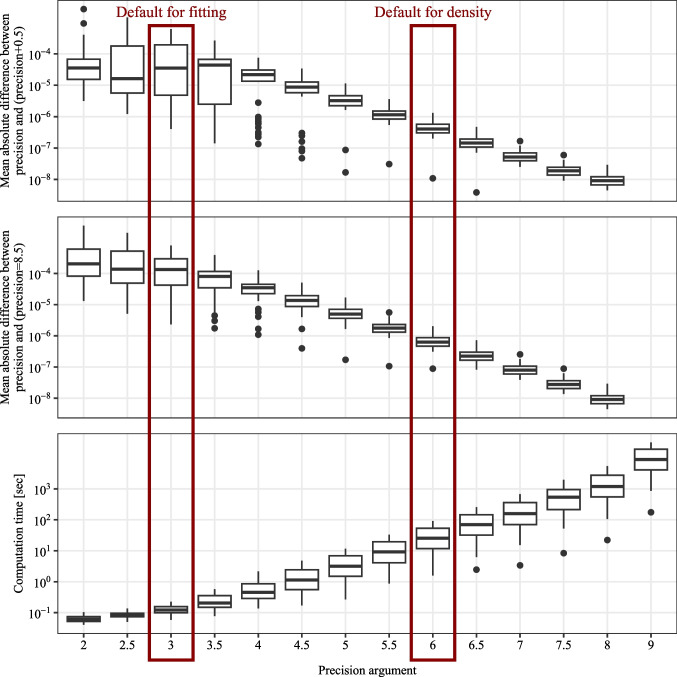
*First Row*: Distribution of mean absolute differences in computed densities between different choices of the precision argument and the argument +0.5. *Second Row*: Distribution of mean absolute differences in computed densities between different choices of the precision argument and the computed densities for precision = 8.5. *Third Row*: Distribution of computation time of the densities for a vector of 600 observations (log scaled). Each observation is based on 600 simulated trials for a random parameter set (50 trials for each combination of discriminability condition and stimulus category)

Note that this is the time to fit each participant if no parallelization is used within the participants. The results suggest a very good model recovery on the individual level (Fig. 11, upper panel) for all models but MTLNR. Race models and DDM-based models can be clearly separated, and within the model architectures, there is only slight confusion among individual subjects. The fitting procedure misidentifies the MTLNR with the 2DSD in 13 out of the 50 simulated data sets (26%), which indicates that there is a high risk of model mimicry between these two models. At the group level, the PEP for each generative model was close to 1 across the 50 data sets, with MTLNR achieving the lowest PEP (.9991) and values indistinguishable from 1 for the other models. Because experimental studies with a large number of trials often rely on smaller sample sizes, we conducted a bootstrap analysis, drawing 1000 subsamples of ten data sets for each generative model and computing PEP on each subset. This analysis is rather conservatives because studies with only ten participants and 500 trials per participant seem rather unreliable in general when using diffusion models and non-hierarchical methods. The results indicated that the model recovery of 2DSD, dynaViTE, IRMt, and PCRMt was robust (Fig. 11, lower panel). The only exception was MTLNR, which was occasionally misidentified as 2DSD. Note that all selection analyses were conducted using the BIC, the most conservative criterion. When using the less conservative AIC, results look similar at the individual level but slightly better at the group level (Supplementary Fig. 23) for the comparison between 2DSD and dynaViTE. The AIC is therefore preferable over the BIC when comparing these two models.

These results indicate that, while comparisons between diffusion-based and race models are very robust in general, both at the individual and group levels, some models may exhibit model mimicry at the individual level and, if the sample size is small, at the group level as well. Group-level comparisons are very robust for a sufficiently large sample size. In cases of small samples and a small number of trials, we therefore recommend conducting a model-mimicry analysis of the fitted models using the parameter estimates for the data at hand.

**Fig. 13:**
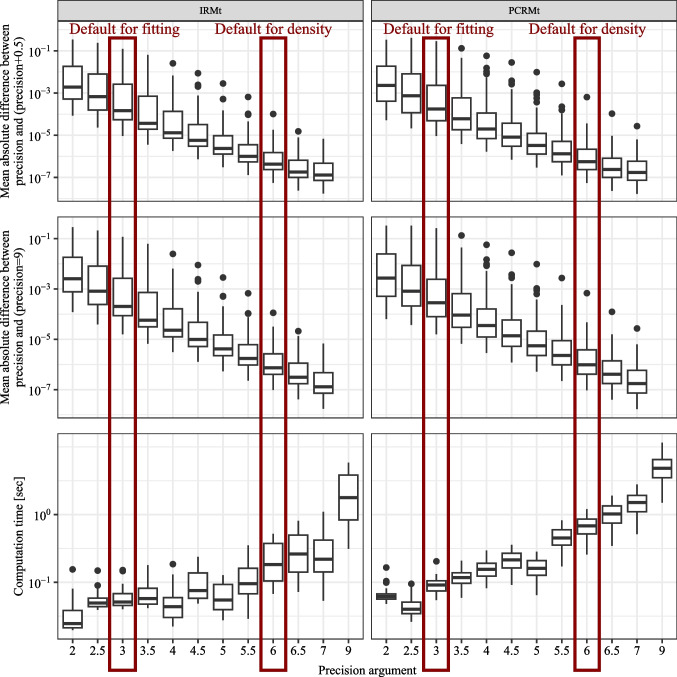
*First Row*: Distribution of mean absolute differences in computed densities between consecutive increases in the exponent of the precision argument. *Second Row*: Distribution of mean absolute differences in computed densities between different choices of the precision argument and the computed densities for precision = 9. *Third Row*: Distribution of computation time of the densities for a vector of 600 observations (log scaled). Each observation is based on 600 simulated trials for a random parameter set (50 trials for each combination of discriminability condition and stimulus category)

## Precision analysis

Two numerical approximations are involved in the computation of the probability densities. First, in the drift-diffusion-based models (dynaViTE, dynWEV, 2DSD, and DDConf), the infinite series in the formula for the first-passage time density is approximated using a truncated summation for which an upper bound for the error is available (Navarro & Fuss, 2009). However, the integration over the variation in the starting point and the non-decision time component is not analytically solvable and is thus computed numerically. This second approximation also introduces uncertainty in the precision of the density computations. The numerical computation of the integral uses a rectangular approximation of the density with an equidistant grid of support points. The step size for this approximation is controlled by the precision argument. The value of the precision argument is transformed into step sizes using similar computations as in the rtdists package (Singmann et al., 2020). Because there are no mathematical guarantees in the form of upper error bounds, we conducted a simulation study to assess the expected error for different values of the precision arguments.

**Fig. 14:**
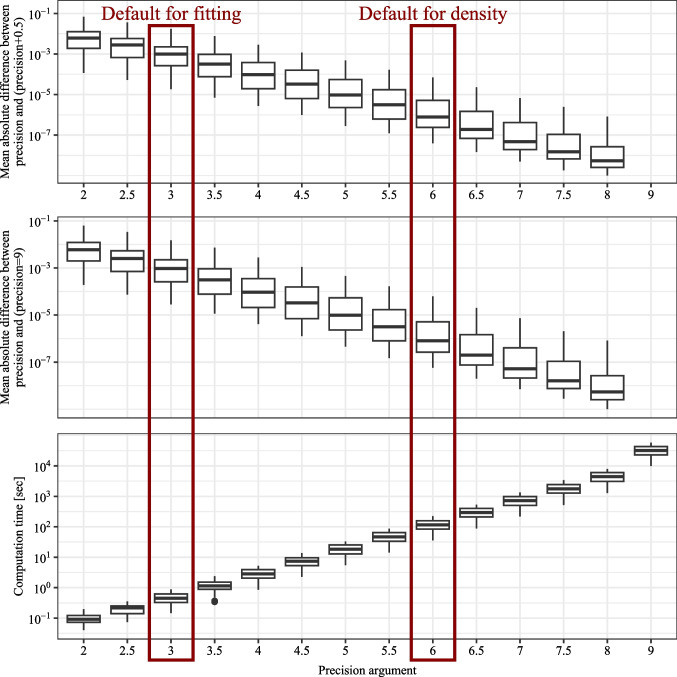
*First Row*: Distribution of mean absolute differences in computed densities between consecutive increases in the exponent of the precision argument. *Second Row*: Distribution of mean absolute differences in computed densities between different choices of the precision argument and the computed densities for precision = 9. *Third Row*: Distribution of computation time of the densities for a vector of 600 observations (log scaled). Each observation is based on 600 simulated trials for a random parameter set (50 trials for each combination of discriminability condition and stimulus category)

### Method

We estimated the expected error by computing the densities to simulated observations from different parameter sets several times with different values for the precision argument. Afterward, we computed the mean difference between the calculated density values as a measure of the computational error. Parameter sets were simulated as follows.

Confidence judgments were assumed to be measured live on a three-point scale (i.e., $K=3$), and there were three levels of stimulus discriminability (i.e., $L=3$). This means that for dynaViTE, there were $11+3+2\cdot 2=18$ parameters, for IRMt and PCRMt, there were $6+3+4=13$ parameters, and for MTLNR, there were $10+3+2\cdot 2=17$ parameters.

Parameters were sampled independently and uniformly, with some exceptions. First, the discriminability parameters were uniformly distributed for the first level, and the differences between discriminability levels were uniformly distributed. This ensured increasing levels of discriminability. Second, starting-point variability in dynaViTE was uniformly distributed across the admissible range, depending on the mean starting-point parameter *z*. Similarly, the three weight parameters $w_X$, $w_{RT}$, and $w_{Int}$ are sampled sequentially. In addition, for the upper decision boundaries of the two processes in race models, their sum and relative height were independently uniformly distributed to more closely resemble how boundary separation and relative starting point in dynaViTE were sampled. Finally, the confidence thresholds were computed based on a simulated proportion of ratings, which ensured a minimum number of observations in each category.

We derived parameter ranges from previously conducted model fits to empirical data from (Hellmann et al., 2023, 2024), and the example in this paper.

We sampled 50 random parameter sets per model for dynaViTE, IRMt, PCRMt, and MTLNR. A data set with 50 trials for each combination of discriminability condition and stimulus category was generated for each parameter set (600 trials per data set in total). Finally, we computed the trial-wise likelihood for the simulated data with different precision arguments. For dynaViTE and MTLNR, we used values from 2 to 8 in steps of 0.5, plus a value of 9. For the race models, we used values from 2 to 7 in steps of 0.5, plus a precision of 9. The probabilities attained with the highest precision, i.e., 9, were used as references for the other precision values. To estimate the absolute error, we computed the mean absolute distance between each precision’s probabilities and the reference. In addition, we computed the mean absolute difference between two consecutive precision values to estimate the expected improvement of the density calculation. For full details, we refer the reader to the analysis code.

### Results

The mean absolute differences for the computed probability densities between different values of precision arguments are depicted for dynaViTE in Fig. 12, for the race models in Fig. 13, and for MTLNR in Fig. 14.

For all models, the estimated error and the difference between subsequent values of the precision argument both start far below the required value for low precision values and then decrease exponentially as functions of the precision argument for higher values. Assuming that the exponential decrease of subsequent differences continues, we can infer that the magnitude of the error in the computed densities is proportional to the estimated error in our simulation. This means that with the default value of 6 for the precision argument in the densities, the expected absolute error is about $10^{-6}$ of magnitude, while for a value of 7.5, it is $10^{-7.5}$. In general, the transformation between the precision argument and the step size used for the numerical integration is chosen such that the provided precision represents the number of digits correctly calculated on average.

Notably, the computation time for the probability densities also increases exponentially with the precision argument (Figs. 12-14, lower rows), which clarifies the trade-off between precision and computation time. Because only one numerical integration is necessary for race models – IRMt, PCRMt, and MTLNR do not require integration for between-trial variability in starting points – the computation time should be expected to be less influenced by the precision. While we see a lower intercept and slope for IRMt and PCRMt, i.e., faster computations, the computation time for MTLNR is similar to the dynaViTE, suggesting that the step-size required for obtaining a required precision is much smaller compared to the other models.

Fitting the parameters to experimental data using the default arguments for the fitting function may require up to 140,000 evaluations of the negative log-likelihood of the data (around 12,000 parameter sets in the initial grid search) plus 125,000 evaluations from the optimization (5 starting parameter sets each with five calls of the optimization routine each with a maximum of 5000 function evaluations per optimization call). Keeping the computation time low is essential for the applicability of these models (see Fig. 9). Therefore, we use a default precision of 3 in the fitting functions. Evaluating the likelihood with 600 trials takes much less than a second. Still, the results from the recovery analyses show that this is sufficient to produce a high model and parameter recovery.

In addition, with the default values, the precision of parameter estimates and likelihoods is often limited by factors such as the timing of stimulus presentation and the measurement precision of reaction times. For experimental response time data, the precision of reaction time measurements is often limited to milliseconds and depends on the hardware and software used to conduct the experiment (Bridges et al., 2020; Plant & Turner, 2009). For online studies, the precision is often lower (Anwyl-Irvine et al., 2021; Semmelmann & Weigelt, 2017).

## Summary

The **dynConfiR** package implements state-of-the-art computational models of choice, response time, and decision confidence based on the drift-diffusion model and race models of choice. The R package may prove an attractive tool for psychology and cognitive neuroscience researchers because it offers a user-friendly implementation of probability distributions for observed data and functions for parameter fitting, prediction, and simulation. The package is freely available from the CRAN repository, providing sustainable access and facilitating its installation for users.

## Contributions and bug reports

Any issues and observed bugs in the package may be reported here: https://github.com/SeHellmann/dynConfiR/issues. Fi-nally, we encourage any contributions in the form of additional implemented models or extensions of the package functionalities in the form of pull requests. A brief instruction on how to contribute new models is available at https://github.com/SeHellmann/dynConfiR/.

## Supplementary Information

Below is the link to the electronic supplementary material.

Supplementary file 1 (pdf 8692 KB)

## Data Availability

Data sets are available for download at https://github.com/SeHellmann/dynConfiR_Paper.

## Code availability

The **dynConfiR** package is available on CRAN (https://cran.r-project.org/web/packages/dynConfiR/). This paper is based on version 1.1.1 of the package. Data and code for this paper are available at https://github.com/SeHellmann/dynConfiR_Paper. The development version of the package is available at https://github.com/SeHellmann/dynConfiR

## Declarations

### Code availability

Code for all analyses and production of figures is available for download at https://github.com/SeHellmann/dynConfiR_Paper.
