# Supplementary material for: dynConfiR: An R package for sequential sampling models of decision confidence
Source: Behav Res Methods. 2026 May 8;58(6):159. doi: 10.3758/s13428-026-03013-0 (PMC13156207; doi:10.3758/s13428-026-03013-0)
Supplement: Supplementary file 1 — (pdf 8692 KB) [file 13428_2026_3013_MOESM1_ESM.pdf]

Supplementary material for
dynConfiR: An R package for sequential sampling models of decision confidence

Sebastian Hellmann, Michael Zehetleitner, Manuel Rausch

April 2026

1 Mathematical Details of the Multiple Threshold Log-Normal Race Model

The boundary crossing times $T_i = D_i/V_i$ are marginally distributed according to a log-normal distribution with mean parameter $\mu_i = \mu_{di} - \mu_{vi}$ and variance parameter $\sigma_i^2 = \sigma_{di}^2 + \sigma_{vi}^2$. The joint distribution of both boundary crossing times (T_1, T_2) is a multivariate log-normal distribution with mean parameter (μ_1, μ_2) and covariance matrix:

$$\Sigma = \begin{pmatrix} \sigma_1^2 & \sigma_1\sigma_2\rho \\ \sigma_1\sigma_2\rho & \sigma_2^2 \end{pmatrix},$$

with $\rho := \frac{\sigma_{d1}\sigma_{d2}\rho_d + \sigma_{v1}\sigma_{v2}\rho_v}{\sigma_1\sigma_2}$.

This means that in the general formulation, the model has 10 parameters: (μ_{d1}, μ_{d2}, μ_{v1}, μ_{v2}, ρ_d, ρ_v, σ_{d1}, σ_{d2}, σ_{v1}, σ_{v2}). However, the joint distribution of boundary crossing times can be formulated using only four parameters: $(\mu_1, \mu_2, \sigma_1, \sigma_2, \rho)$ as defined above. Therefore, the full set of 10 parameters is not identifiable if only data for a single set of parameters are available. Concerning the mean parameters, the trade-off between μ_{di} and μ_{vi} is directly visible, which is why setting either pair of mean parameters (either for the accumulation rates or boundary distances) is one straight-forward solution to constrain the parameter vector, which is implemented in `dynConfiR`.

However, when it comes to the variance parameters, the constraints to put on the model are not so straightforward. Consider only the marginal variance of T_i. To keep σ_1 constant when changing, e.g., $\sigma'_{d1} := k\sigma_{d1}$ with $k > 0$, we could set $\sigma'_{v1} = \sqrt{\sigma_{d1}^2 + \sigma_{v1}^2 - k^2\sigma_{d1}^2}$. This is only possible, as long as the term under the square root is positive. For this reason, fixing any variance parameter of the original parameter set to a constant, e.g., $\sigma_{d1} = c \in \mathbb{R}_+$, would lead to the marginal variance of the corresponding decision time to be constraint by $\sigma_1^2 = \sigma_{d1}^2 + \sigma_{v1}^2 \geq c$, which restricts the predicted outcome distributions for observed data.

Indeed, the least restrictive simplification in terms of possible predicted distributions is to assume that boundary distances (or accumulation rates) are not varying but constants, e.g., equal to 1. Although this is conceptually a simpler model, the mathematical distribution that such a model can produce is equally flexible as the complete model. Therefore, for simple applications, we would recommend setting $\sigma_{d1} = \sigma_{d2} = \rho_d = \mu_{d1} = \mu_{d2} = 0$ to make the parameters identifiable, but this should also depend on the situation at hand and the experimental manipulations involved.

1.1 Identifiability of MTLNR when fitting with `fitRTConf`

When fitted on data from an experiment with a single discriminability manipulation in `fitRTConf`, we make following assumptions, which are in line with the assumptions made in the other implemented models:

1. Boundary distance parameters $\mu_{d1}, \mu_{d2}, \sigma_{d1}, \sigma_{d2}, \rho_d$ are constant across experimental manipulations.
2. Variance parameters of the accumulation rate $\sigma_{v1}, \sigma_{v2}, \rho_v$ are constant across experimental manipulations.
3. The mean accumulation rate of the correct (matching) accumulator varies in discrete steps between conditions $(\mu_1, \ldots, \mu_K)$, while the mean accumulation rate for the incorrect (non-matching) parameter is constant μ_0 over conditions.

Based on these assumptions, the variance and covariance parameters of the joint distribution of boundary crossing times is constant across conditions. Therefore, we can—without loss of generality—apply the above described simplifications, and label the variance parameters σ_1, σ_2, and ρ. Next, let μ_{d1} and μ_{d2} for now be free parameters to capture possible response biases towards one choice option, similar to diffusion based models that have a free parameter z for the starting point bias. We will consider the mean parameters across different experimental conditions $S \in \{-1, 1\}$, $d \in \{1, 2, ..., K\}$. Assume that the matching accumulator for $S = 1$ is the first and for $S = -1$ is the second accumulator. Then, we mean parameters (μ_{v1}, μ_{v2}) are summarized as follows:

	$S = 1$	$S = -1$
$d = 1$	$(\mu_1 - \mu_{d1}, \mu_0 - \mu_{d2})$	$(\mu_0 - \mu_{d1}, \mu_1 - \mu_{d2})$
$d = 2$	$(\mu_2 - \mu_{d1}, \mu_0 - \mu_{d2})$	$(\mu_0 - \mu_{d1}, \mu_2 - \mu_{d2})$
...	...	...
$d = K$	$(\mu_K - \mu_{d1}, \mu_0 - \mu_{d2})$	$(\mu_0 - \mu_{d1}, \mu_K - \mu_{d2})$

Here, we see that we could subtract μ_0 from all mean parameters and would get the same accumulation rate parameters in all conditions. This is why we chose to set $\mu_0 = 0$ in the fitting procedure.

1.2 Reasoning behind the choice of the internal confidence variable

The idea of multiple thresholds on an accumulator determining confidence is equivalent to confidence being based on the ratio of (predicted) boundary crossing times (see Suppl. Fig. 1).

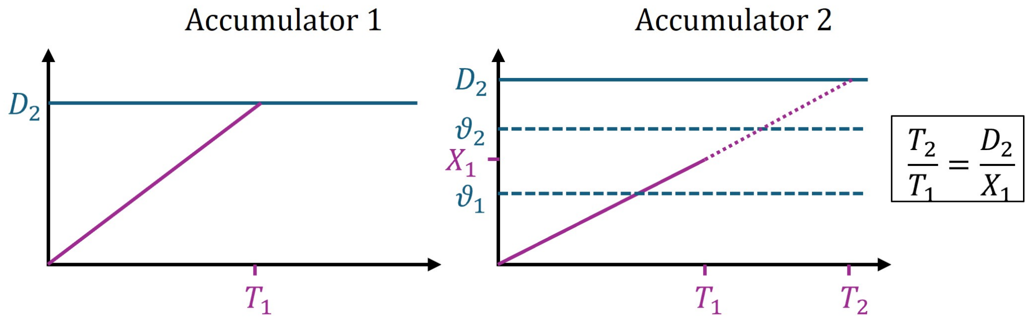

Supplementary Figure 1: Illustration of confidence generation in the MTLNR. The hypothesis of multiple thresholds on the accumulated evidence of one accumulator can be interpreted as thresholds on the ratio of boundary crossing times.

Mathematically, consider the event

$$E := \{T_1 \text{ wins } \& \ X_2 \in [\vartheta_1, \vartheta_2]\}, \qquad \text{with } \vartheta_1 < \vartheta_2 < D_2.$$

The thresholds are considered proportions of the required evidence to trigger a decision (Reynolds et al., 2020). (Having absolutely defined thresholds would cause problems with the log-normally distributed boundary distance.) Therefore, let $\vartheta_i = p_i D_2$, for $0 < p_1 < p_2 < 1$ and $X_2 = pD_2$, for some p. Now, we can relate X_2, directly with the accumulation rate V_2, and T_1, which leads to $pD_2 = X_2 = V_2 T_1 \Leftrightarrow p = \frac{T_1}{D_2/V_2}$. We can interpret the denominator as the hypothetical boundary crossing time of the loosing accumulator. Finally, the event E can be re-formulated to:

$$E = \{T_1 \text{ wins } \& \ T_2/T_1 \in [1/p_2, 1/p_1]\},$$

so we can equivalently base confidence on the differences in log-boundary crossing times with multiple thresholds $\vartheta_1, \vartheta_2 \in (0, \infty)$:

$$E = \{T_1 \text{ wins } \& \ log\,(T_2/T_1) \in [\vartheta_1, \vartheta_2]\}.$$

In accordance with the other confidence models, we label $log\,(T_2/T_1)$ as the internal confidence variable. Using the results in Reynolds et al. (2020), the probability of the first accumulator reaching the threshold first at time T_1, and the difference in log-boundary times being in a certain interval $c_{MTLNR} = \log T_2 -$

$\log T_1 \in [\vartheta_1, \vartheta_2]$ is given by

$$P(T_1, c_{MTLNR} \in [\vartheta_1, \vartheta_2]) = \frac{1}{T_1 \sigma_1 \sqrt{2\pi}} \exp\left[-\frac{(\log T_1 - \mu_{v1})^2}{2\sigma_1^2}\right]$$

$$\left[\Phi\left(\frac{\vartheta_2 + \log T_1 - (\mu_{v2} + \rho\frac{\sigma_2}{\sigma_1}(\log T_1 - \mu_{v1}))}{\sqrt{(1-\rho^2)\sigma_2}}\right)\right.$$

$$\left. - \Phi\left(\frac{\vartheta_1 + \log T_1 - (\mu_{v2} + \rho\frac{\sigma_2}{\sigma_1}(\log T_1 - \mu_{v1}))}{\sqrt{(1-\rho^2)\sigma_2}}\right)\right].$$

2 Example of Application in Model Comparison

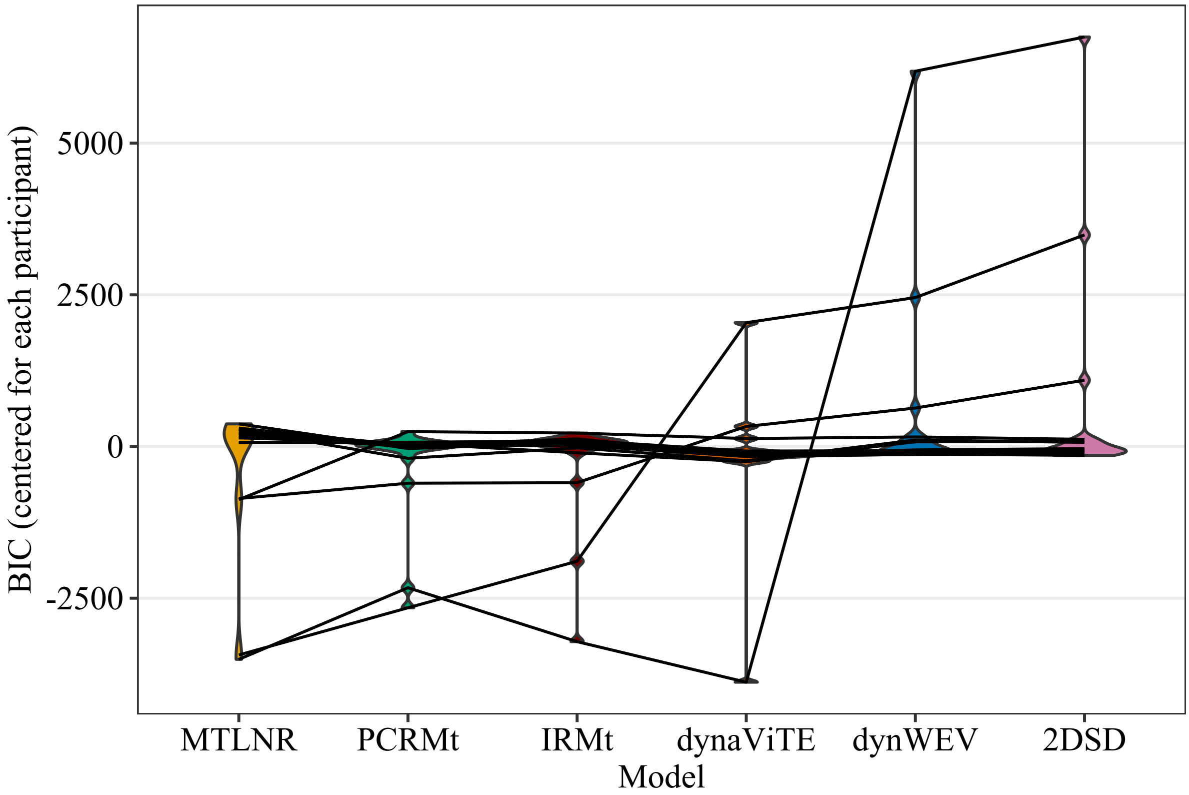

Supplementary Figure 2: Distribution of BIC values, centered for each participant, across different models. Each line represents a participant.

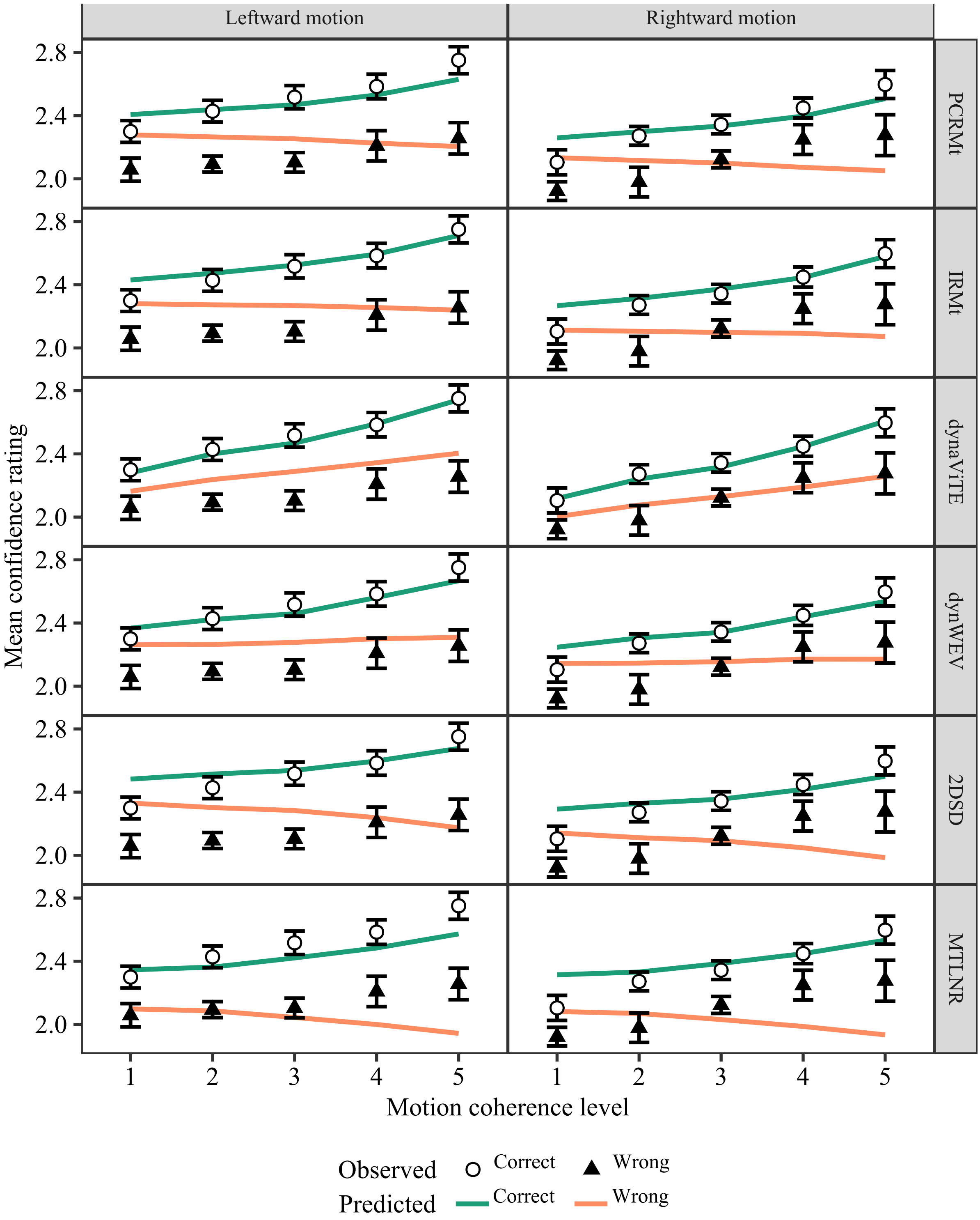

Supplementary Figure 3: Observed (points) and fitted (lines) mean confidence judgments by accuracy (line color, shape) and different responses (columns). Error bars represent within-subject standard errors.

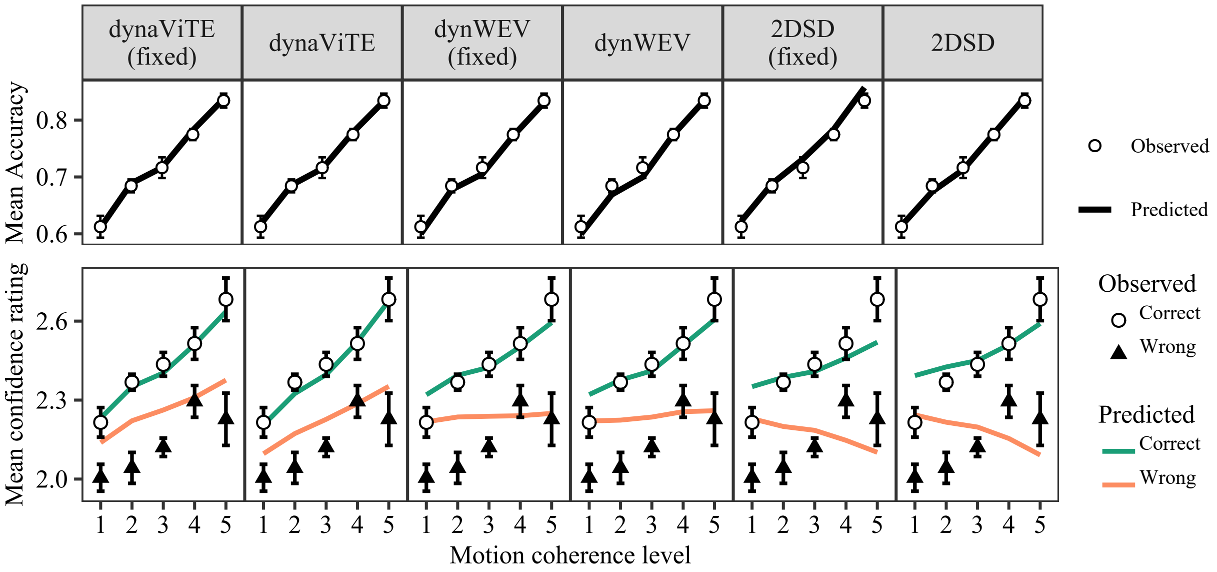

Supplementary Figure 4: Accuracy (top row) and mean confidence rating (bottom row) for empirical data (points and triangles) and model predictions from the full and restricted DDM-based models (lines). Error bars represent within-subject standard errors.

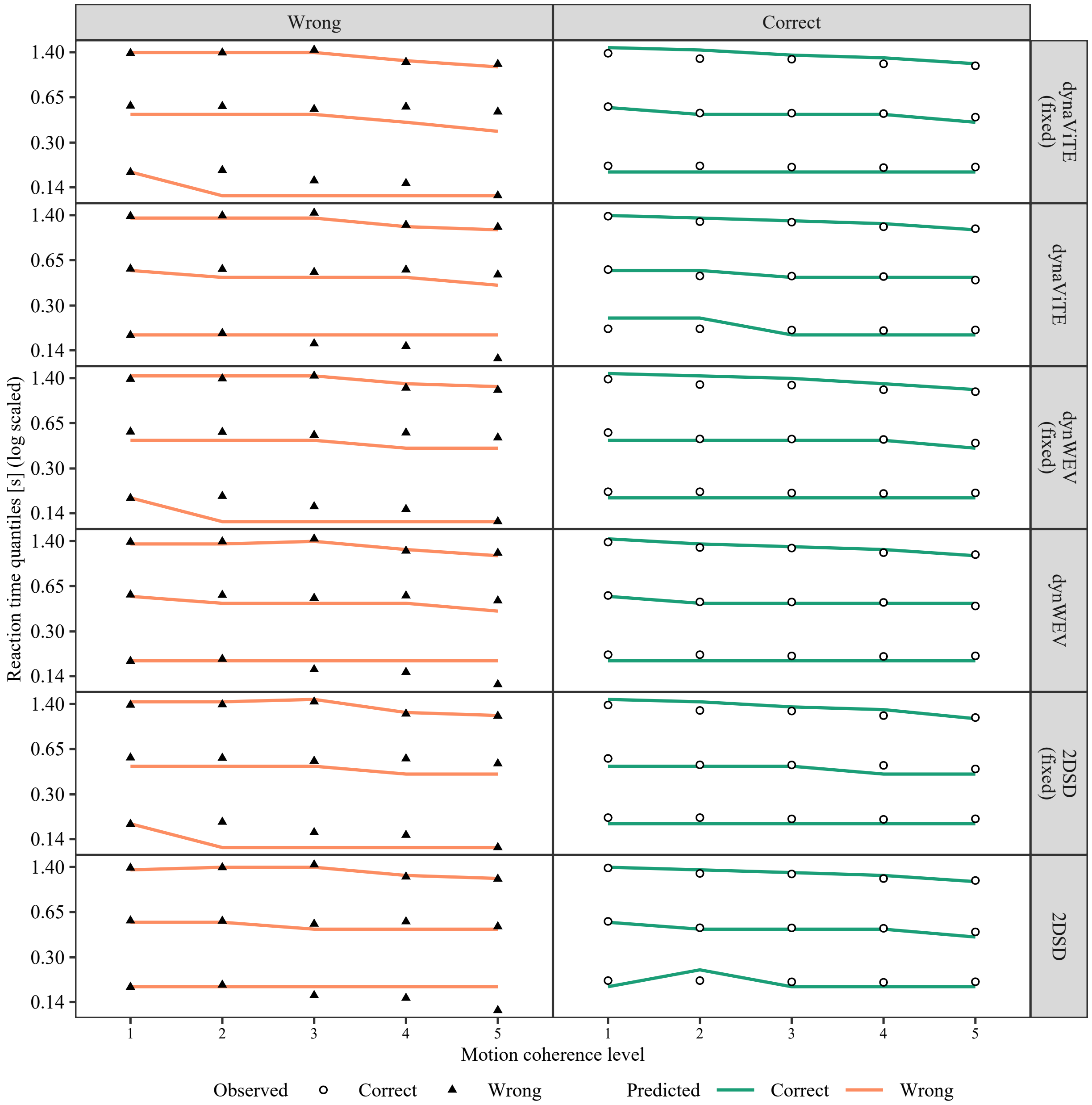

Supplementary Figure 5: Response time quantiles for observed (points) and predicted (lines) response time distributions across correct and incorrect decisions (columns) and levels of stimulus discriminability (x-axis). Probabilities for quantiles: .1, .5, .9.

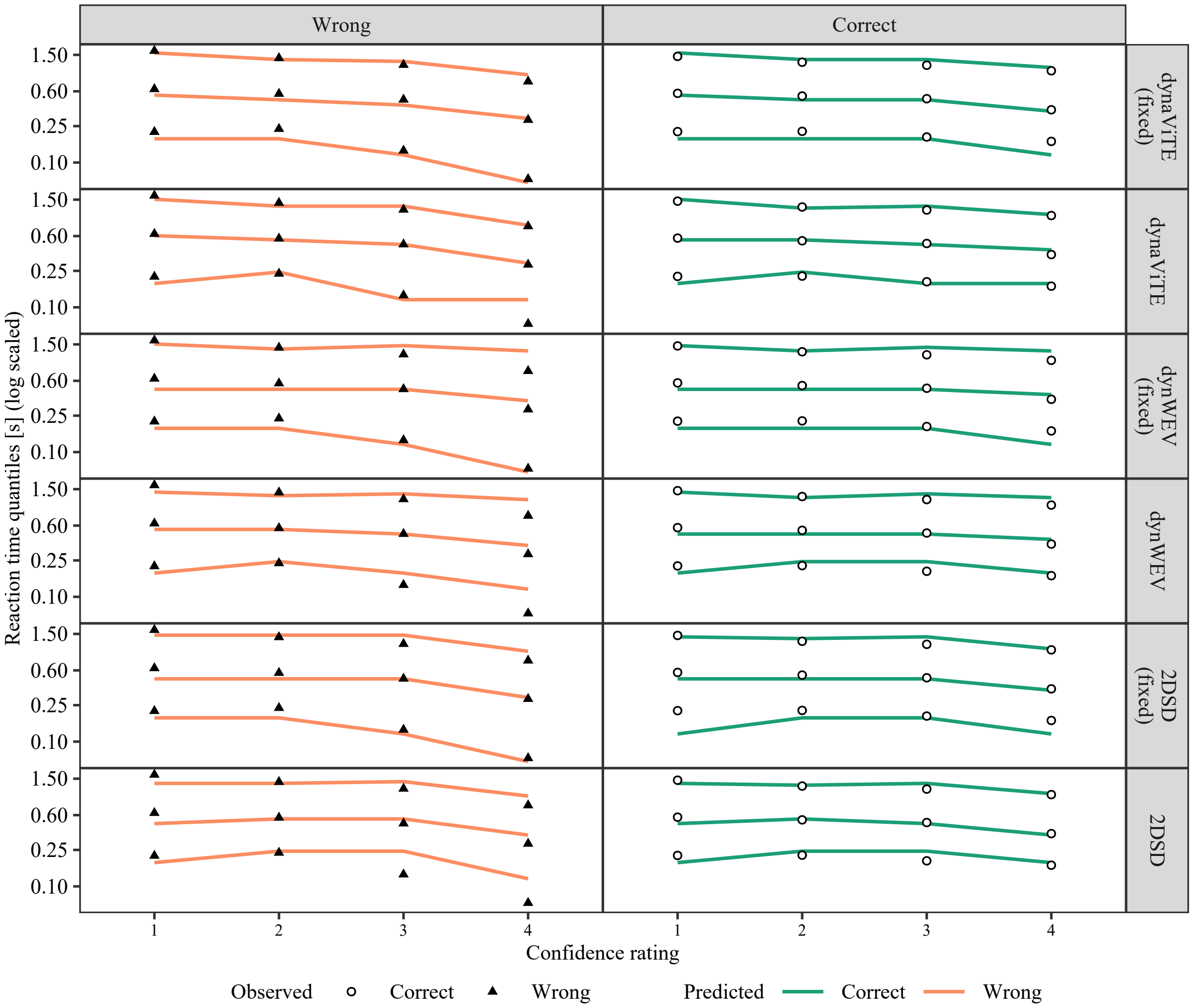

Supplementary Figure 6: Response time quantiles for observed (points) and predicted (lines) response time distributions across correct and incorrect decisions (columns) and confidence ratings (x-axis). Probabilities for quantiles: .1, .5, .9.

3 Parameter Recovery

3.1 Detailed Method

For measuring parameter recovery, we generated artificial data sets from a known set of model parameters, fitted the model to the synthetic data, and compared the recovered parameters to the generating parameters.

We assumed that there were five levels of confidence (i.e., $K = 5$), and there were five levels of stimulus discriminability (i.e., $L = 5$). This means that for dynaViTE, there were $11 + 5 + 2 \cdot 5 = 26$ fitted parameters, for IRMt and PCRMt, there were $6 + 5 + 10 = 21$ parameters, and for MTLRN, there were $7 + 5 + 10 = 22$ parameters.

The generating parameter sets (except for the confidence thresholds) were sampled from parameters derived from previously conducted model fits to empirical data.

We gathered parameter sets from Hellmann et al. (2023, 2024), and the example in this paper. For all four models we used the estimates to the data from Hellmann et al. (2023) and the estimates to the data from Law and Lee (Ng et al., 2021). In addition, dynaViTE, PCRMt, and MTLNR were additionally fitted to the data from Shekhar and Rahnev (2021, see the example in this article). Finally, for MTLNR, we also used estimates to the data from Experiment 2 in Orchard et al. (2022). In total, this resulted in 93 parameter sets for dynaViTE and PCRMt, 73 parameter sets for IRMt, and 110 parameter sets for MTLNR. For the 20 parameter sets fitted to the data of Shekhar and Rahnev (2021), which had only three experimental conditions, the means of the estimated drift rates from two consecutive conditions were used as additional experimental conditions (i.e. the fitted drift rates (ν_1, ν_2, ν_3) were mapped to $(\nu_1, (\nu_1 + \nu_2)/2, \nu_2, (\nu_2 + \nu_3)/2\nu_3))$. For the estimates to the data from Orchard et al. (2022), which had eight difficulty levels, we used the sensitivity parameters of the third and sixth level as sensitivity parameters for the second and fourth level in the simulation. In addition, we computed the average of the first and second, the fourth and fifth, and the seventh and eighth level for the first, third, and fifth level in the simulation, respectively. For more details, see the accompanying code.

Concerning the confidence thresholds, we opted not to utilize the thresholds from the previous model fits. The reason for not using the fitted confidence thresholds was that some participants did not use all confidence categories, resulting in some thresholds being either fitted to plus or minus infinity or coinciding with one another. In contrast, we used the fact that when simulating artificial data, we can simulate the continuous internal confidence variable in the model and compute the confidence thresholds as quantiles of the internal confidence variable, given the proportions of confidence ratings. We fitted a Dirichlet distribution to the observed proportions of confidence ratings from the empirical data of all participants. We then drew random probability vectors from the Dirichlet distribution as proportions of confidence reports, resampling if any proportion was less than 2%. The confidence thresholds were then computed as quantiles of the internal confidence variable in the simulated data set. This procedure ensured that each confidence level contained a non-zero proportion of responses.

To assess the number of trials necessary to recover the parameters, we sampled either 50, 100, 200, or 500 trials per condition and stimulus identity. For two stimulus identities and five levels of discriminability, this leads to 500, 1,000, 2,000, and 5,000 trials per simulated data set for five discriminability conditions.

For each number of artificial trials, we sampled 100 parameter sets and generated one data set per parameter set.

To measure parameter recovery performance, we computed the concordance correlation coefficient (CCC; Lin, 1989), which, in contrast to Pearson correlation, is reduced by non-zero intercepts and non-unit slopes. Therefore, in contrast to Pearson's correlation coefficient, it is sensitive to deviations from the identity line.

3.2 Recovery Plots

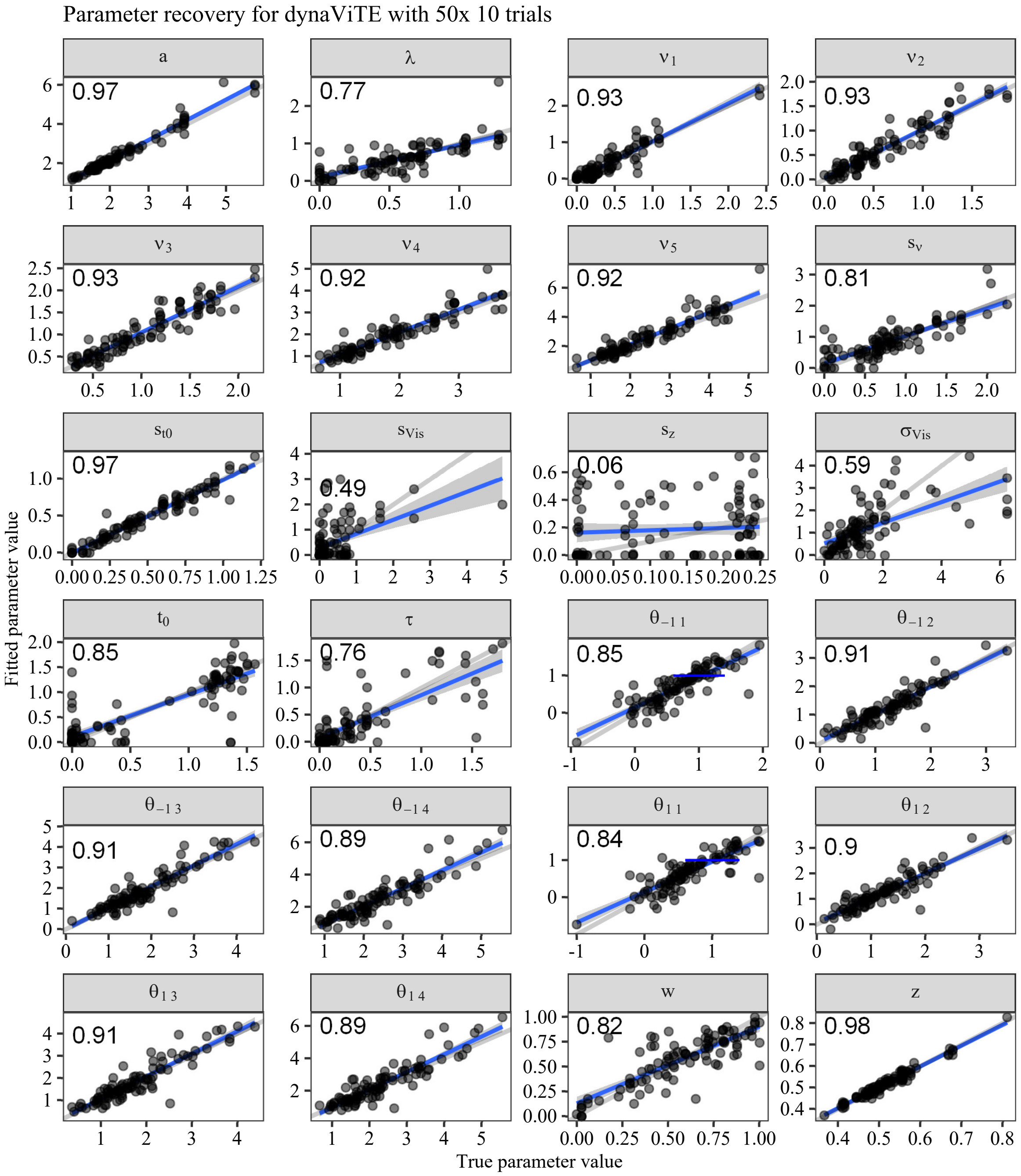

Supplementary Figure 7: Results from parameter recovery analysis for dynaViTE with 50 trials per condition and stimulus identity. Recovered vs. true generative parameters across parameters. Each point represents one simulated parameter and data set. The blue line and shaded area show a linear regression line with 95% confidence band. The grey line shows the identity line. Numbers in the panels show the concordance correlation coefficient for the parameter.

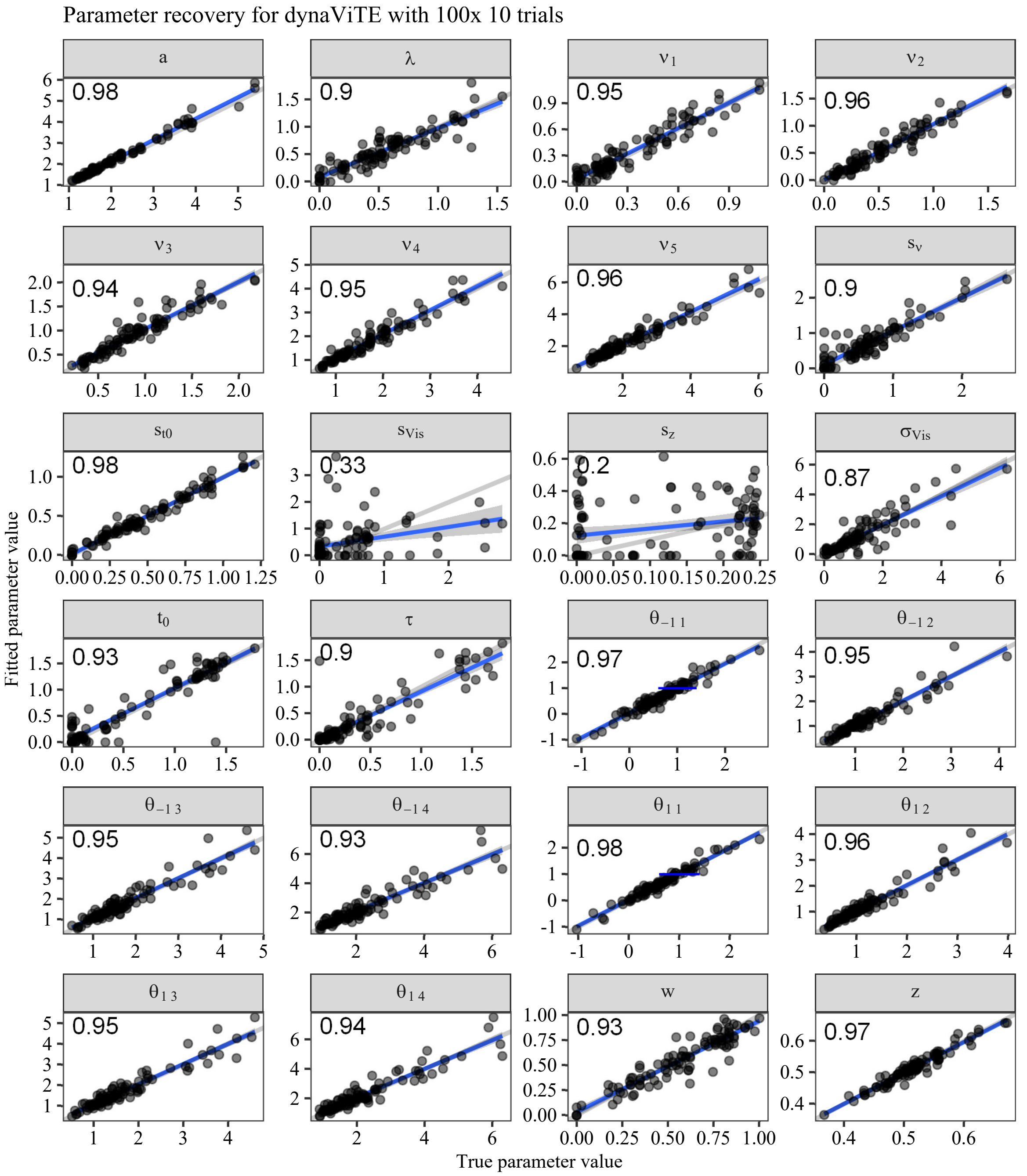

Supplementary Figure 8: Results from parameter recovery analysis for dynaViTE with 100 trials per condition and stimulus identity. Recovered vs. true generative parameters across parameters. Each point represents one simulated parameter and data set. The blue line and shaded area show a linear regression line with 95% confidence band. The grey line shows the identity line. Numbers in the panels show the concordance correlation coefficient for the parameter.

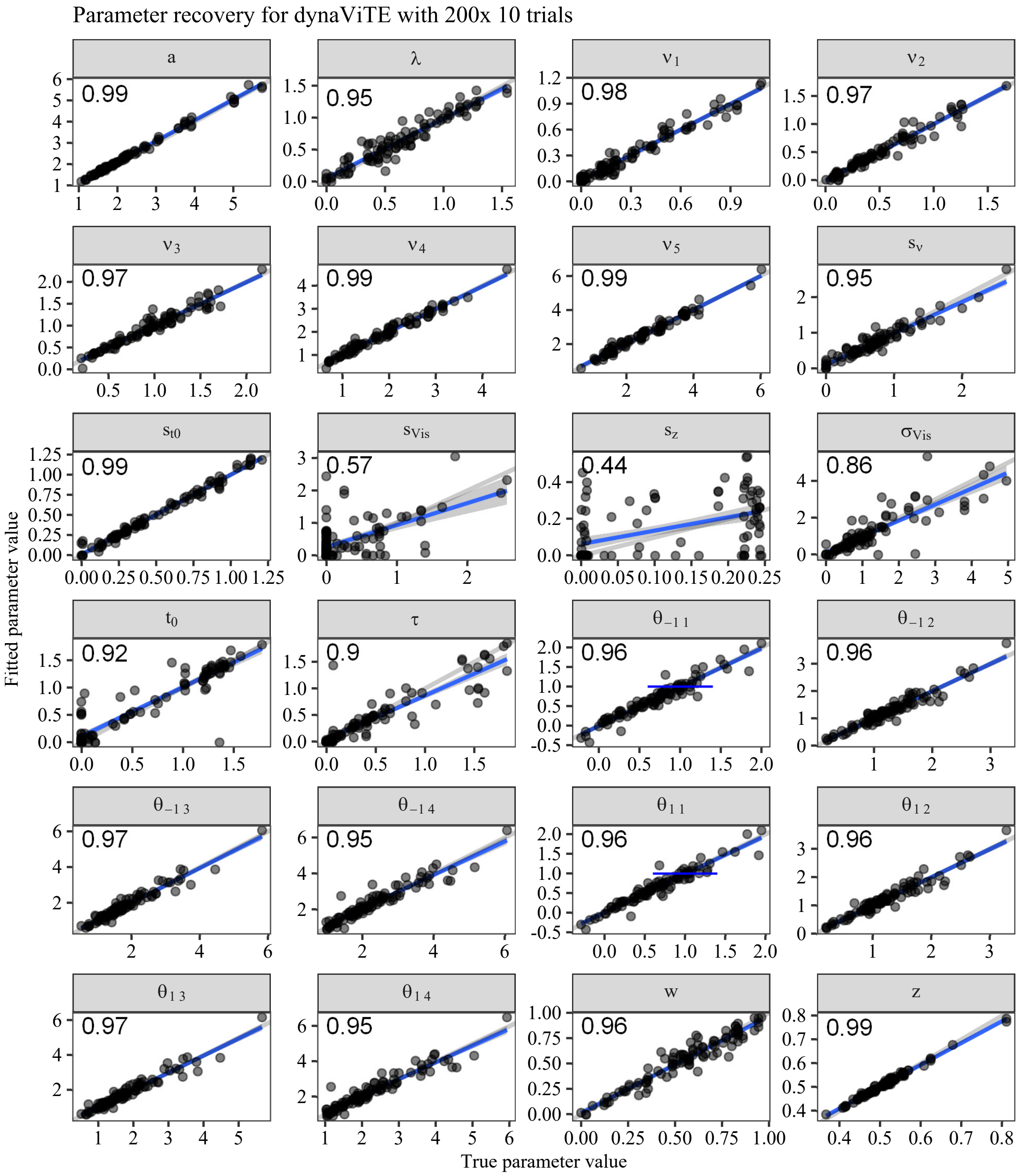

Supplementary Figure 9: Results from parameter recovery analysis for dynaViTE with 200 trials per condition and stimulus identity. Recovered vs. true generative parameters across parameters. Each point represents one simulated parameter and data set. The blue line and shaded area show a linear regression line with 95% confidence band. The grey line shows the identity line. Numbers in the panels show the concordance correlation coefficient for the parameter.

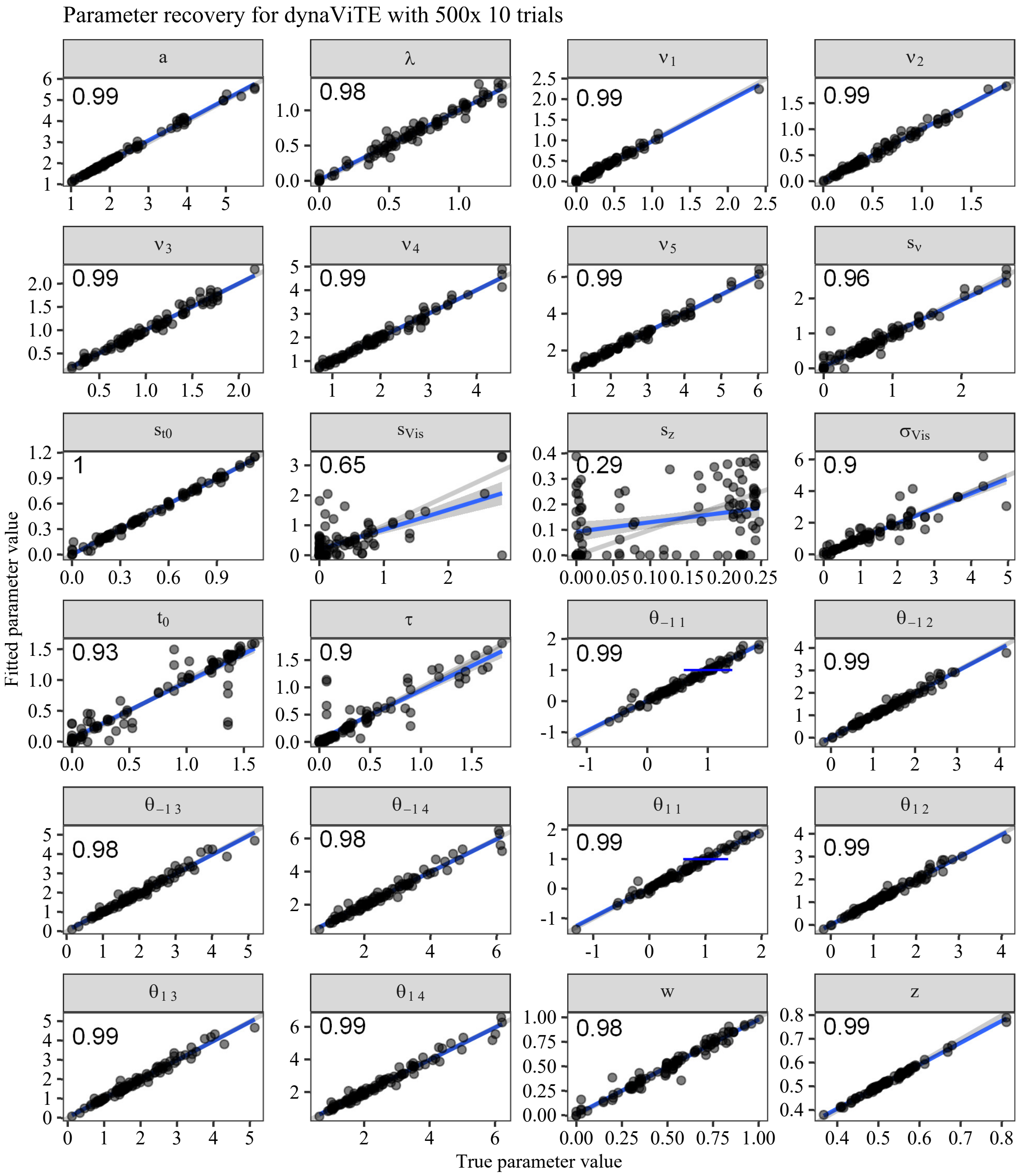

Supplementary Figure 10: Results from parameter recovery analysis for dynaViTE with 500 trials per condition and stimulus identity. Recovered vs. true generative parameters across parameters. Each point represents one simulated parameter and data set. The blue line and shaded area show a linear regression line with 95% confidence band. The grey line shows the identity line. Numbers in the panels show the concordance correlation coefficient for the parameter.

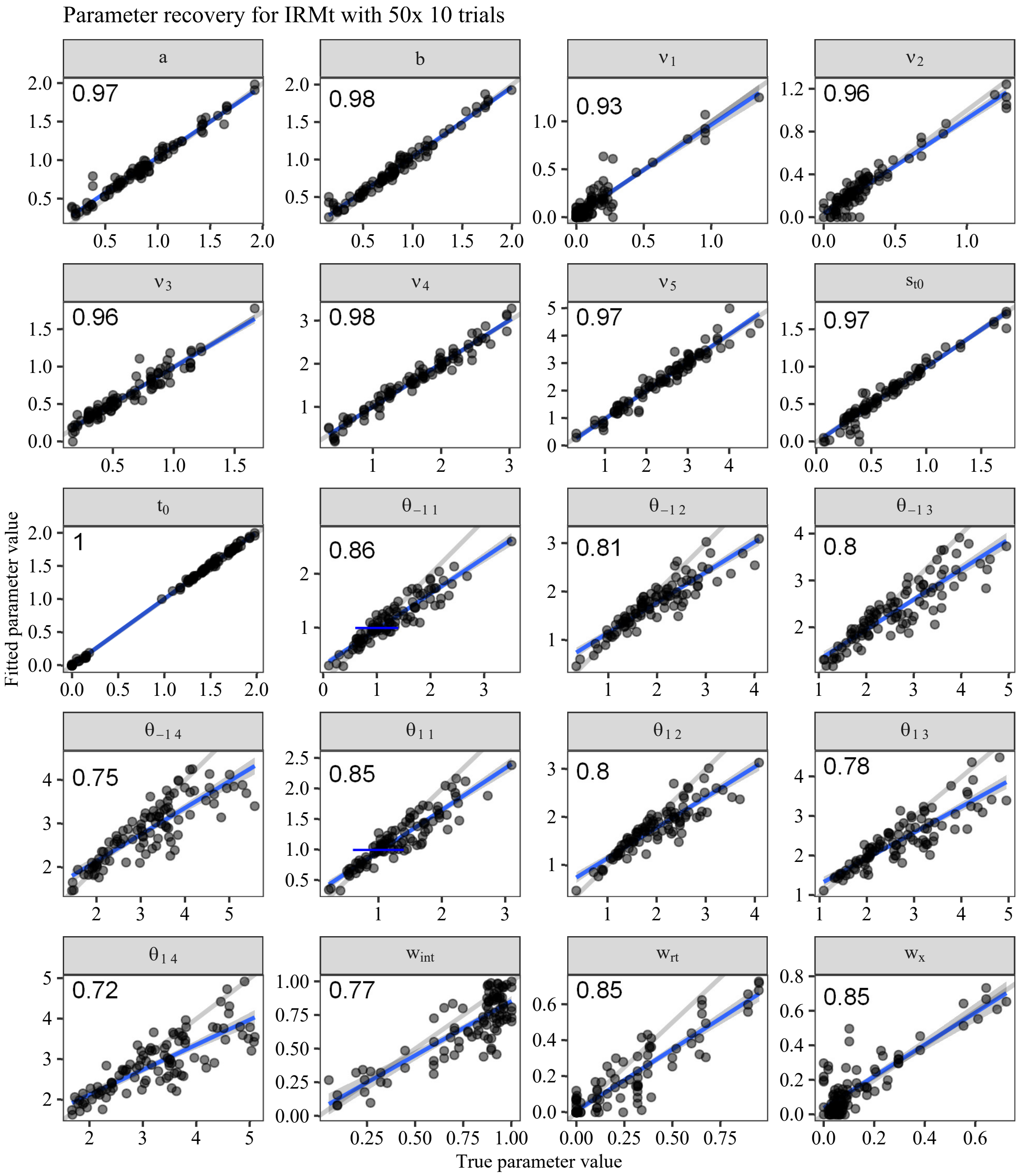

Supplementary Figure 11: Results from parameter recovery analysis for IRMtwith 50 trials per condition and stimulus identity. Recovered vs. true generative parameters across parameters. Each point represents one simulated parameter and data set. The blue line and shaded area show a linear regression line with 95% confidence band. The grey line shows the identity line. Numbers in the panels show the concordance correlation coefficient for the parameter.

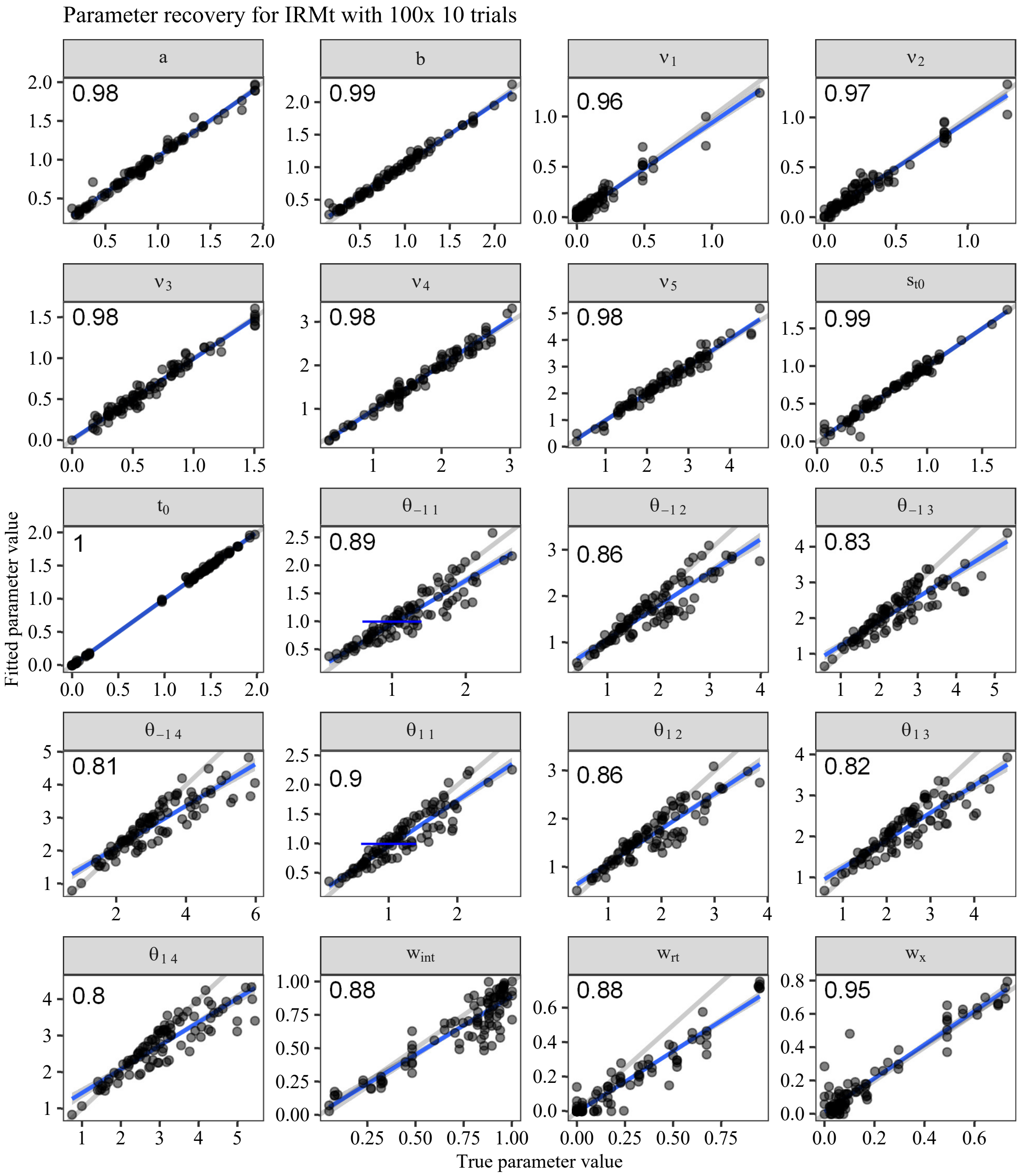

Supplementary Figure 12: Results from parameter recovery analysis for IRMtwith 100 trials per condition and stimulus identity. Recovered vs. true generative parameters across parameters. Each point represents one simulated parameter and data set. The blue line and shaded area show a linear regression line with 95% confidence band. The grey line shows the identity line. Numbers in the panels show the concordance correlation coefficient for the parameter.

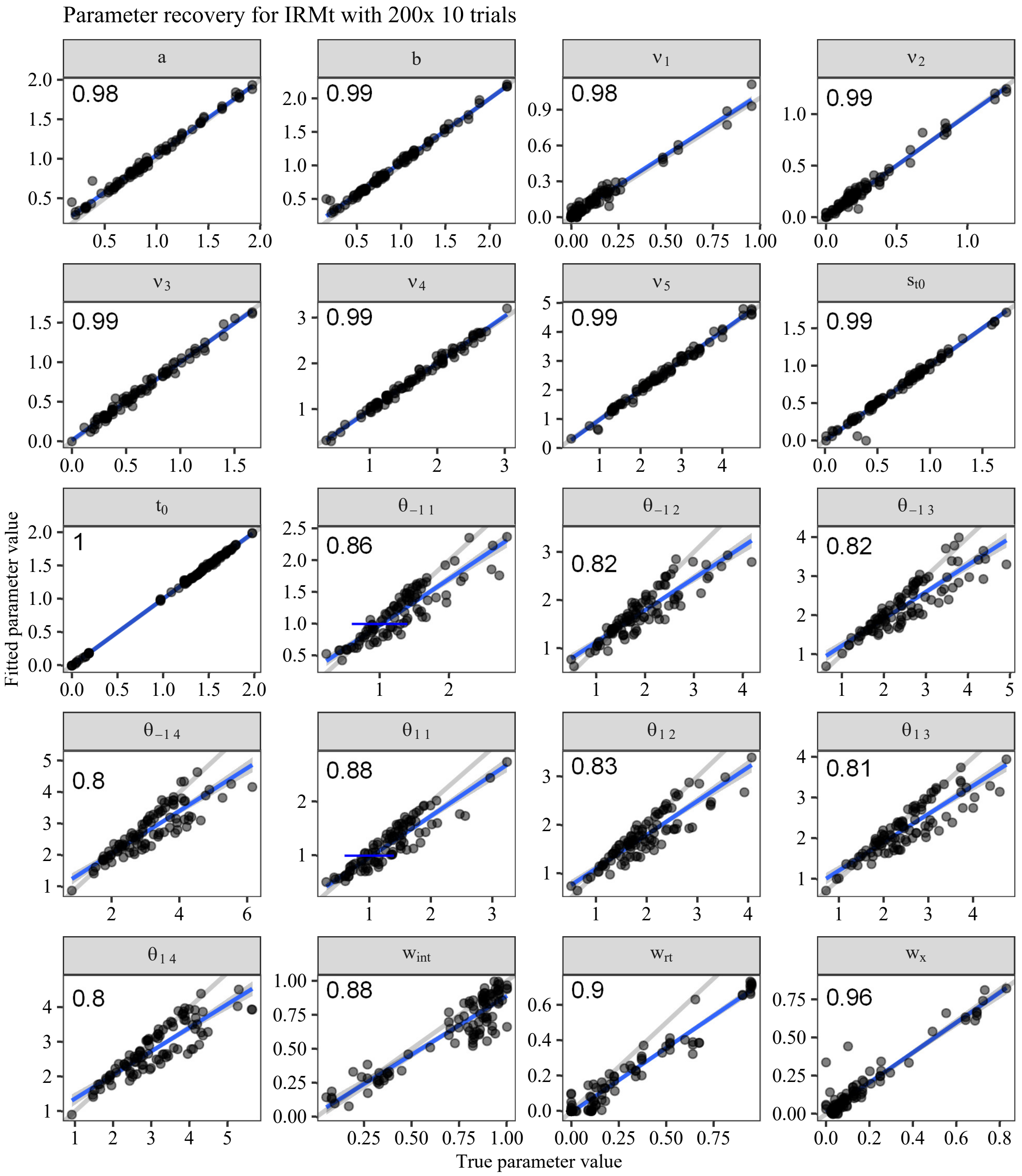

Supplementary Figure 13: Results from parameter recovery analysis for IRMtwith 200 trials per condition and stimulus identity. Recovered vs. true generative parameters across parameters. Each point represents one simulated parameter and data set. The blue line and shaded area show a linear regression line with 95% confidence band. The grey line shows the identity line. Numbers in the panels show the concordance correlation coefficient for the parameter.

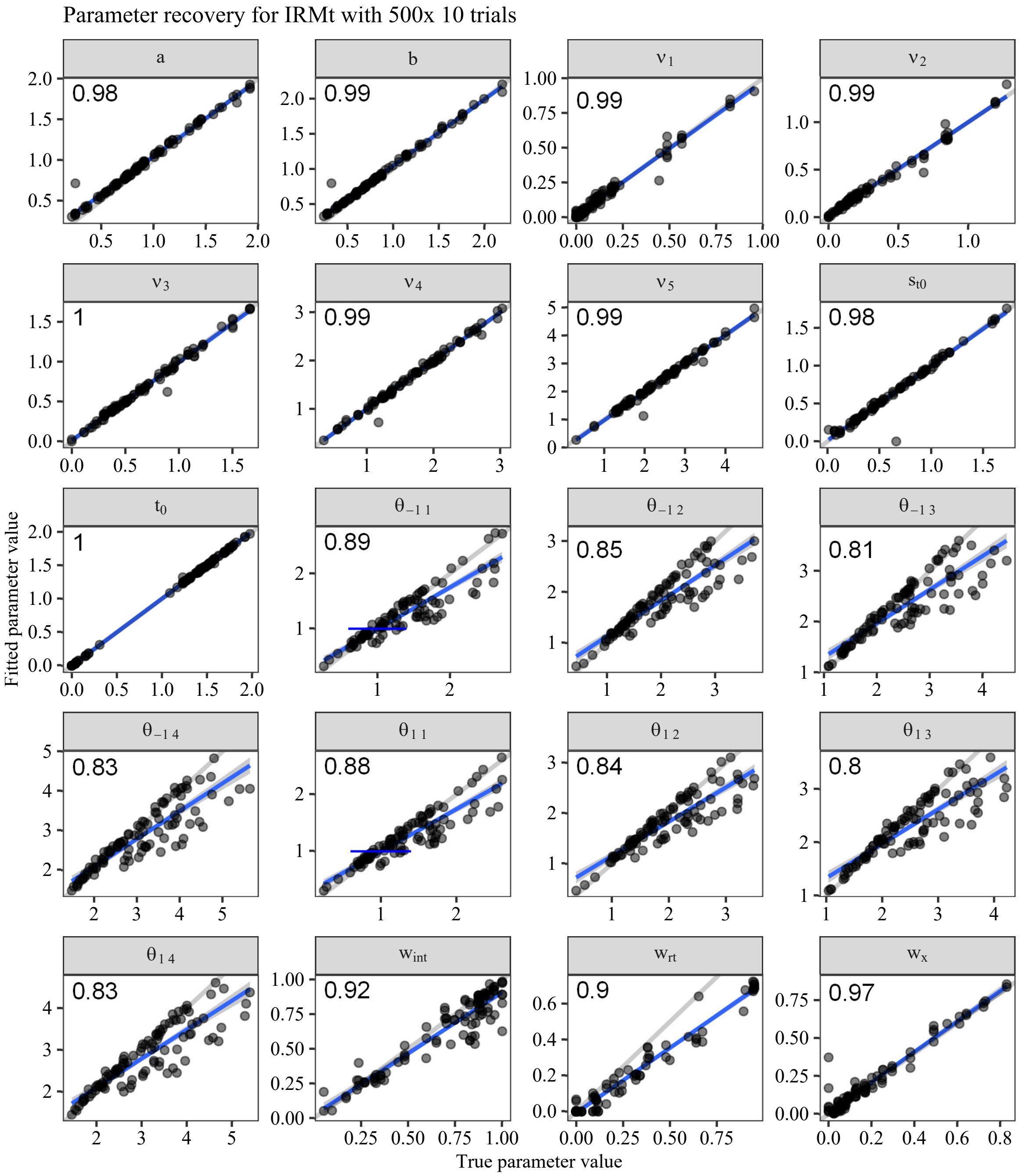

Supplementary Figure 14: Results from parameter recovery analysis for IRMtwith 500 trials per condition and stimulus identity. Recovered vs. true generative parameters across parameters. Each point represents one simulated parameter and data set. The blue line and shaded area show a linear regression line with 95% confidence band. The grey line shows the identity line. Numbers in the panels show the concordance correlation coefficient for the parameter.

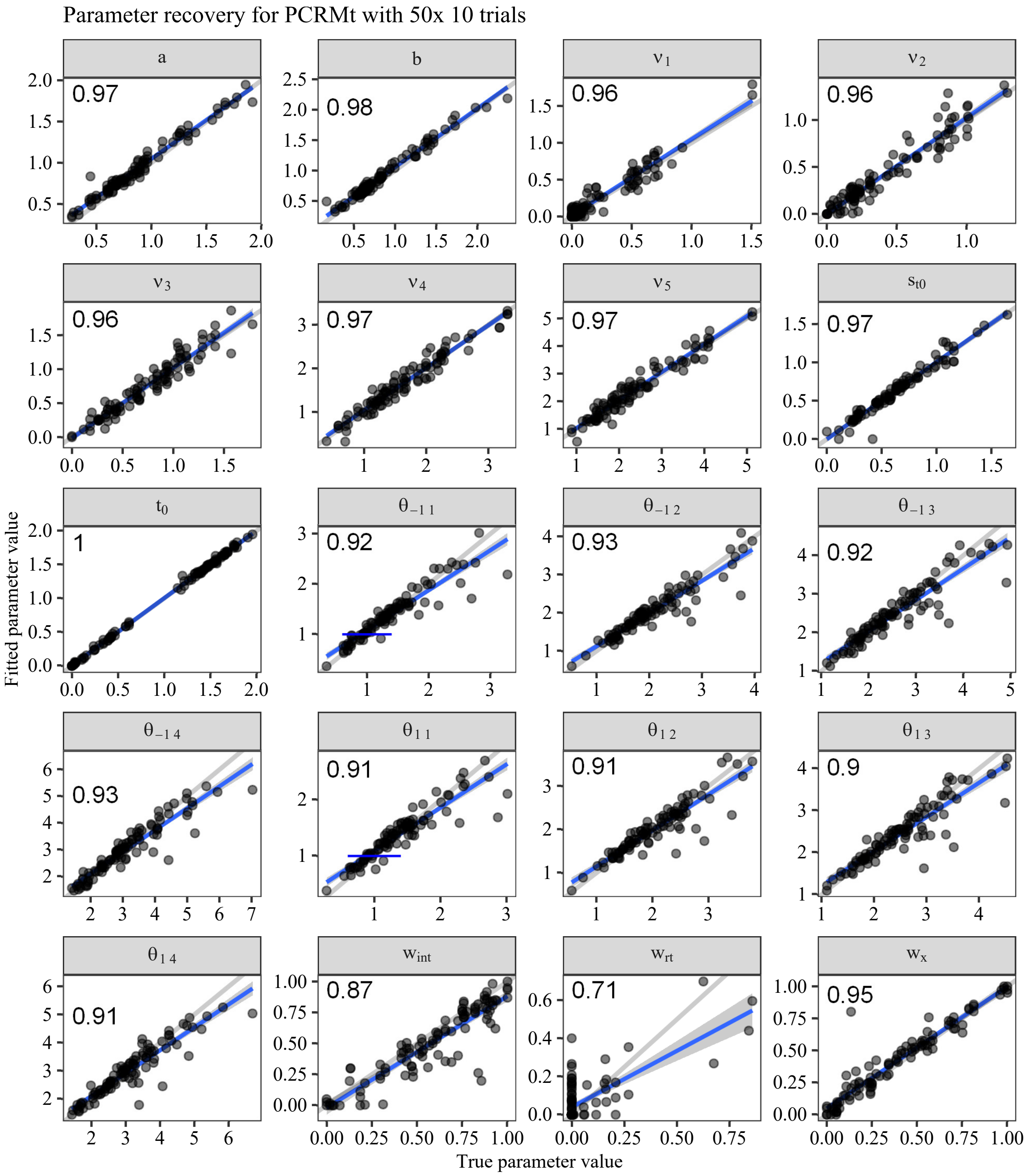

Supplementary Figure 15: Results from parameter recovery analysis for PCRMtwith 50 trials per condition and stimulus identity. Recovered vs. true generative parameters across parameters. Each point represents one simulated parameter and data set. The blue line and shaded area show a linear regression line with 95% confidence band. The grey line shows the identity line. Numbers in the panels show the concordance correlation coefficient for the parameter.

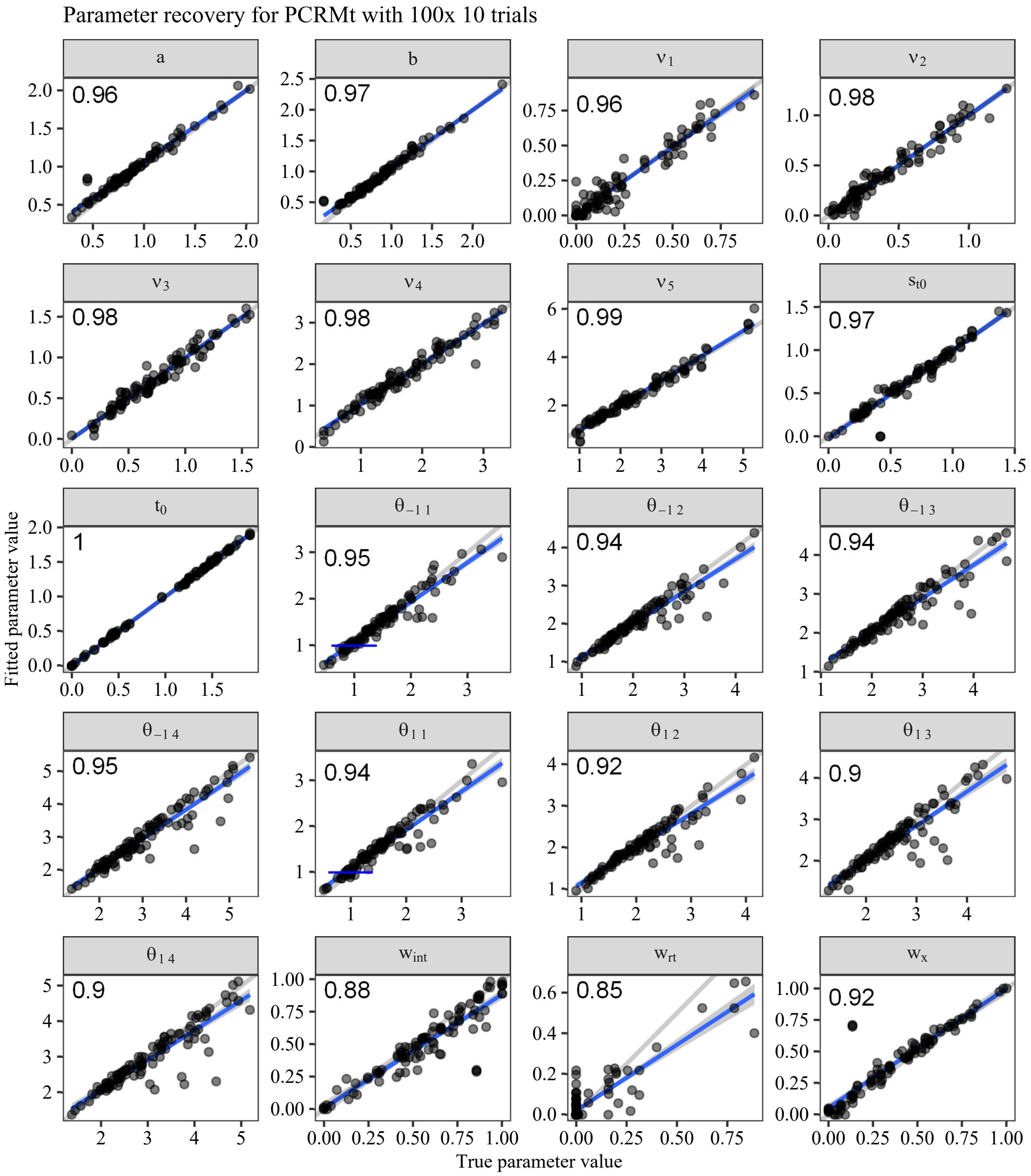

Supplementary Figure 16: Results from parameter recovery analysis for PCRMtwith 100 trials per condition and stimulus identity. Recovered vs. true generative parameters across parameters. Each point represents one simulated parameter and data set. The blue line and shaded area show a linear regression line with 95% confidence band. The grey line shows the identity line. Numbers in the panels show the concordance correlation coefficient for the parameter.

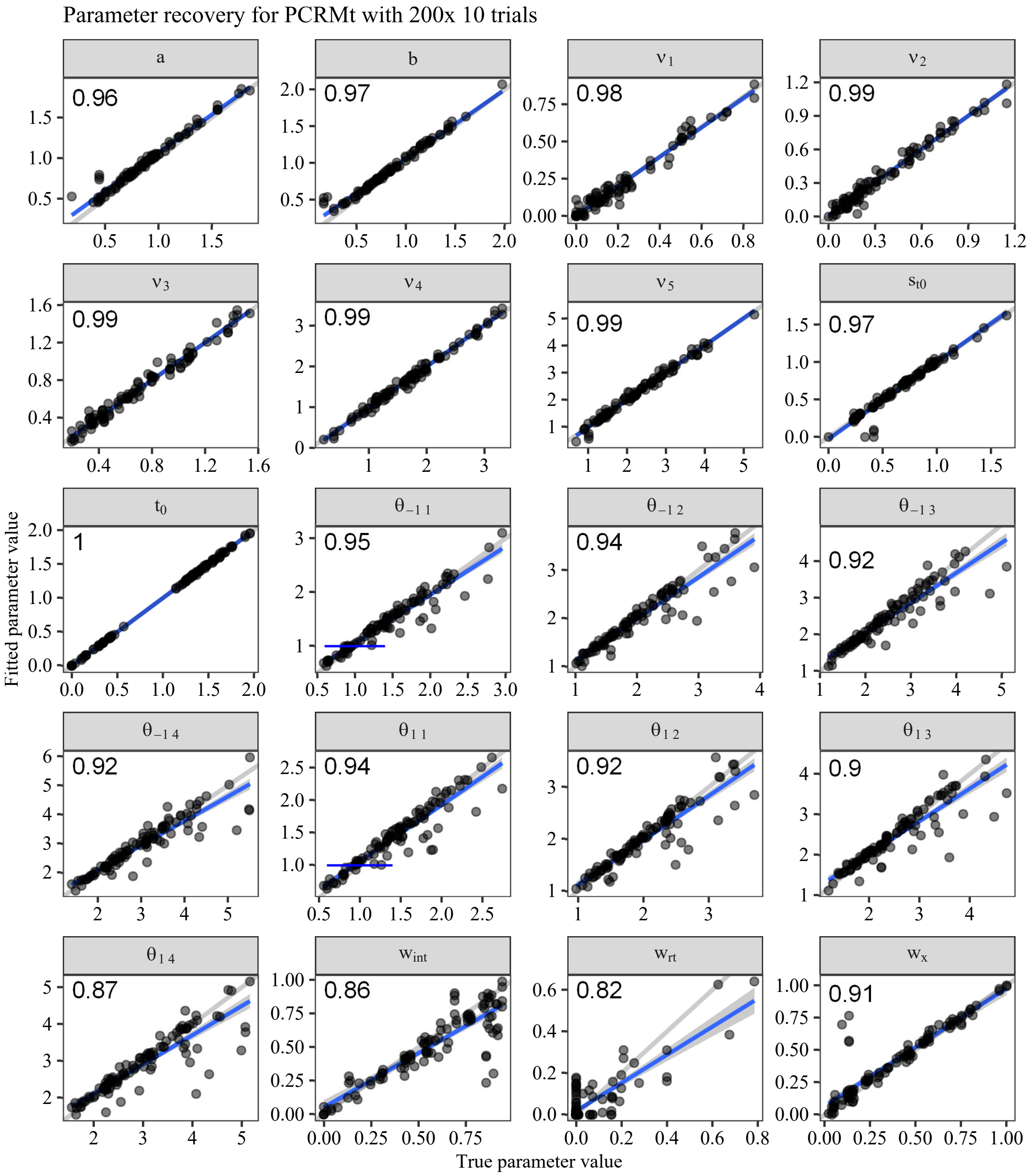

Supplementary Figure 17: Results from parameter recovery analysis for PCRMtwith 200 trials per condition and stimulus identity. Recovered vs. true generative parameters across parameters. Each point represents one simulated parameter and data set. The blue line and shaded area show a linear regression line with 95% confidence band. The grey line shows the identity line. Numbers in the panels show the concordance correlation coefficient for the parameter.

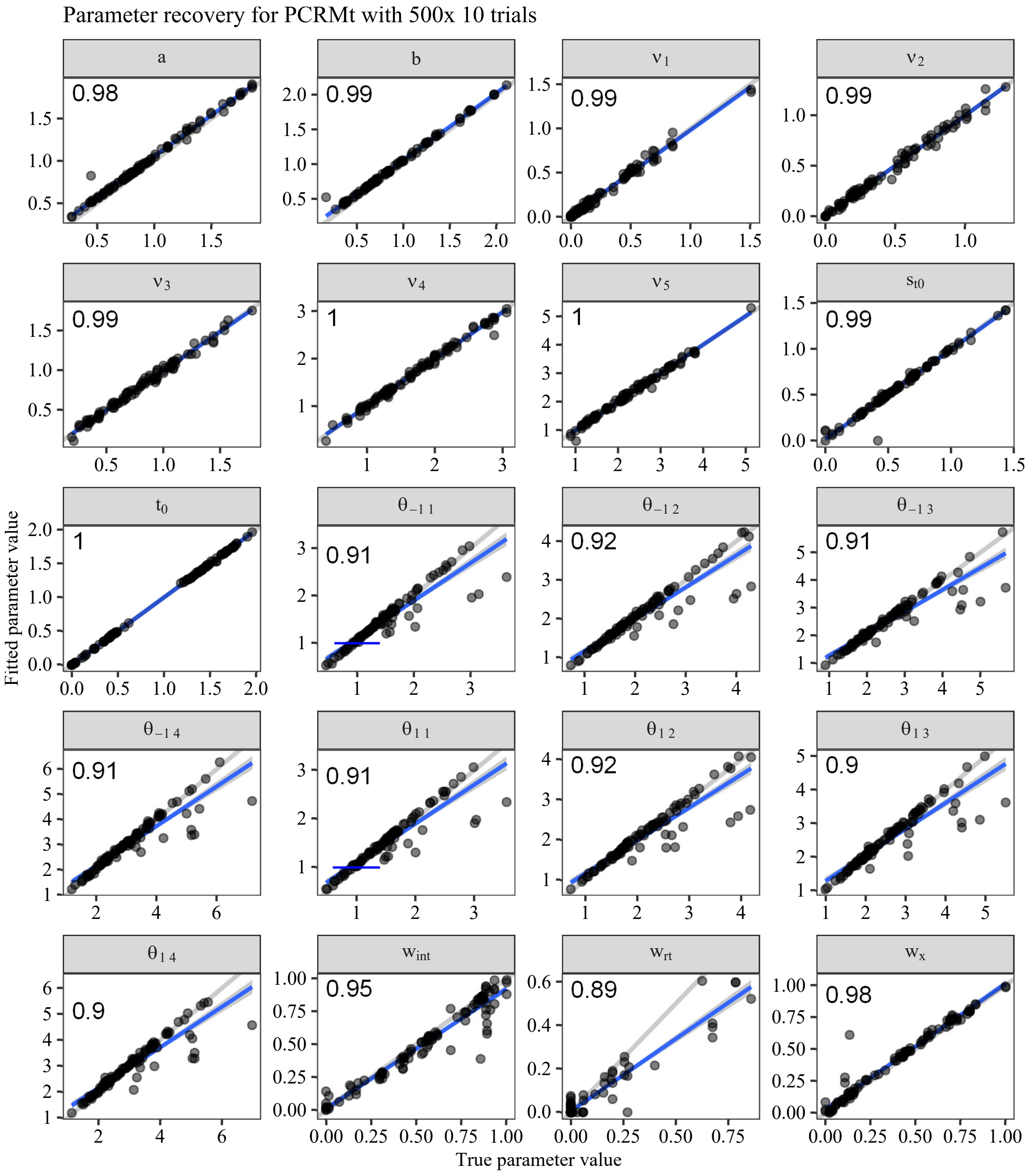

Supplementary Figure 18: Results from parameter recovery analysis for PCRMtwith 500 trials per condition and stimulus identity. Recovered vs. true generative parameters across parameters. Each point represents one simulated parameter and data set. The blue line and shaded area show a linear regression line with 95% confidence band. The grey line shows the identity line. Numbers in the panels show the concordance correlation coefficient for the parameter.

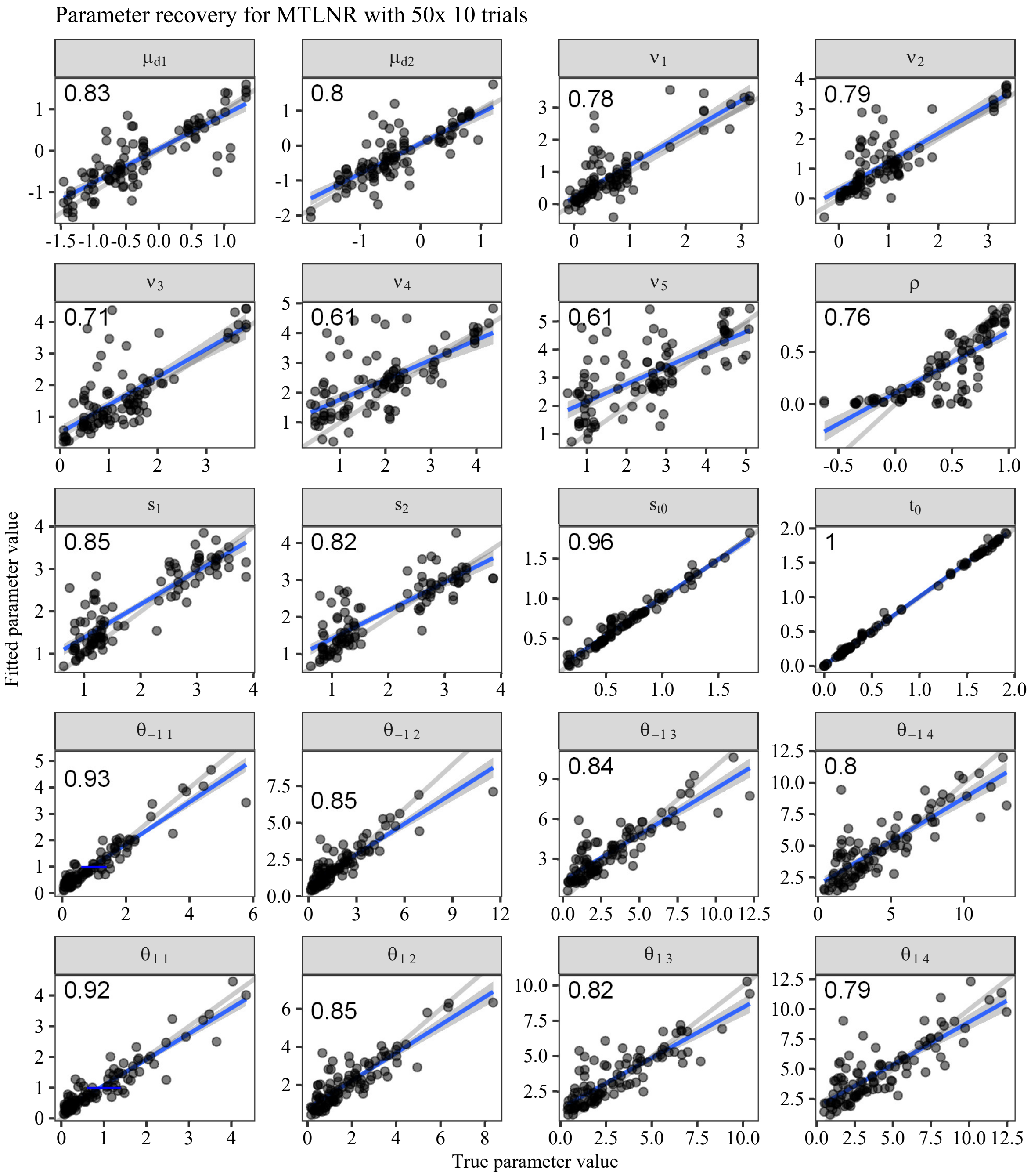

Supplementary Figure 19: Results from parameter recovery analysis for MTLNRwith 50 trials per condition and stimulus identity. Recovered vs. true generative parameters across parameters. Each point represents one simulated parameter and data set. The blue line and shaded area show a linear regression line with 95% confidence band. The grey line shows the identity line. Numbers in the panels show the concordance correlation coefficient for the parameter.

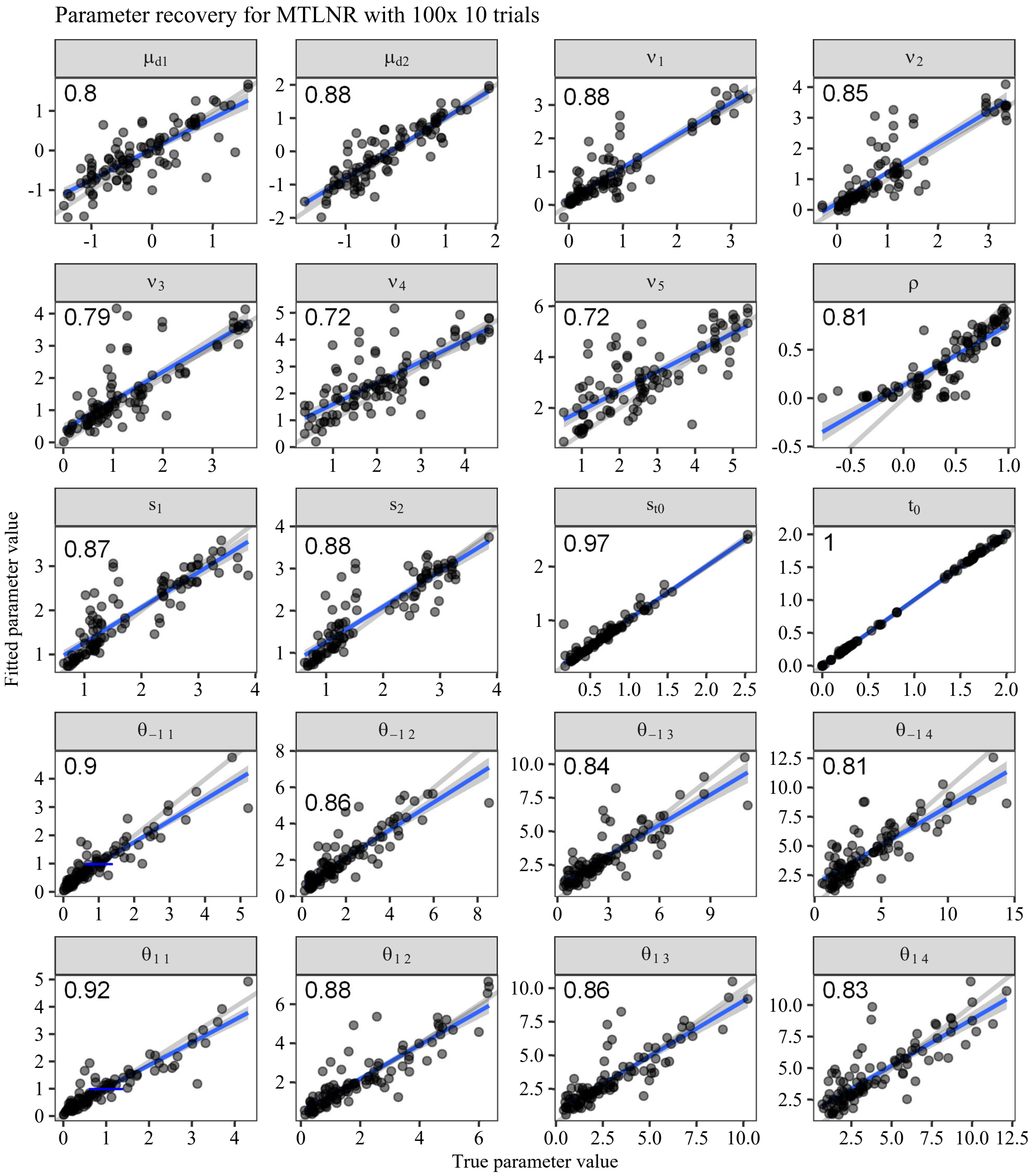

Supplementary Figure 20: Results from parameter recovery analysis for MTLNRwith 100 trials per condition and stimulus identity. Recovered vs. true generative parameters across parameters. Each point represents one simulated parameter and data set. The blue line and shaded area show a linear regression line with 95% confidence band. The grey line shows the identity line. Numbers in the panels show the concordance correlation coefficient for the parameter.

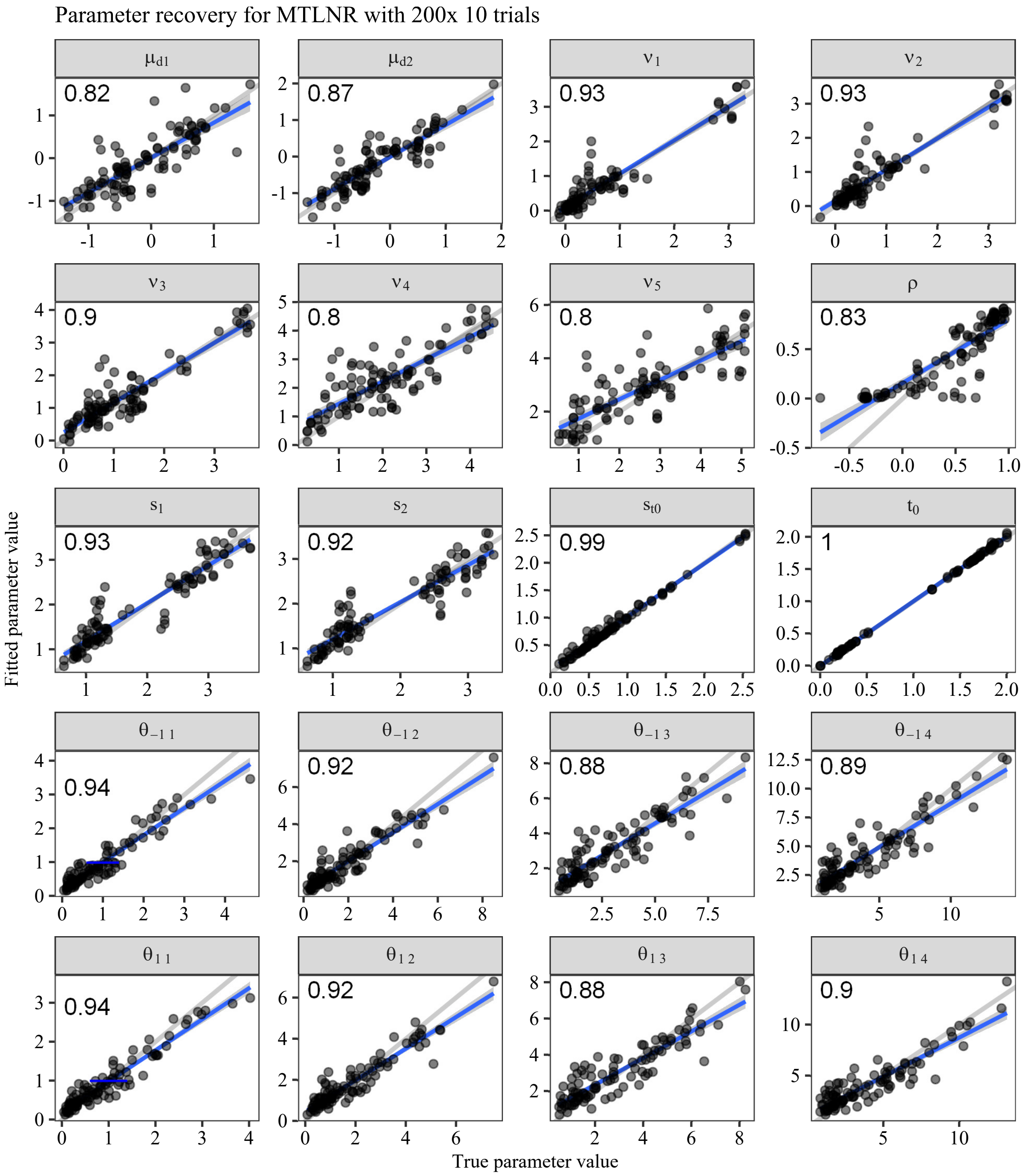

Supplementary Figure 21: Results from parameter recovery analysis for MTLNRwith 200 trials per condition and stimulus identity. Recovered vs. true generative parameters across parameters. Each point represents one simulated parameter and data set. The blue line and shaded area show a linear regression line with 95% confidence band. The grey line shows the identity line. Numbers in the panels show the concordance correlation coefficient for the parameter.

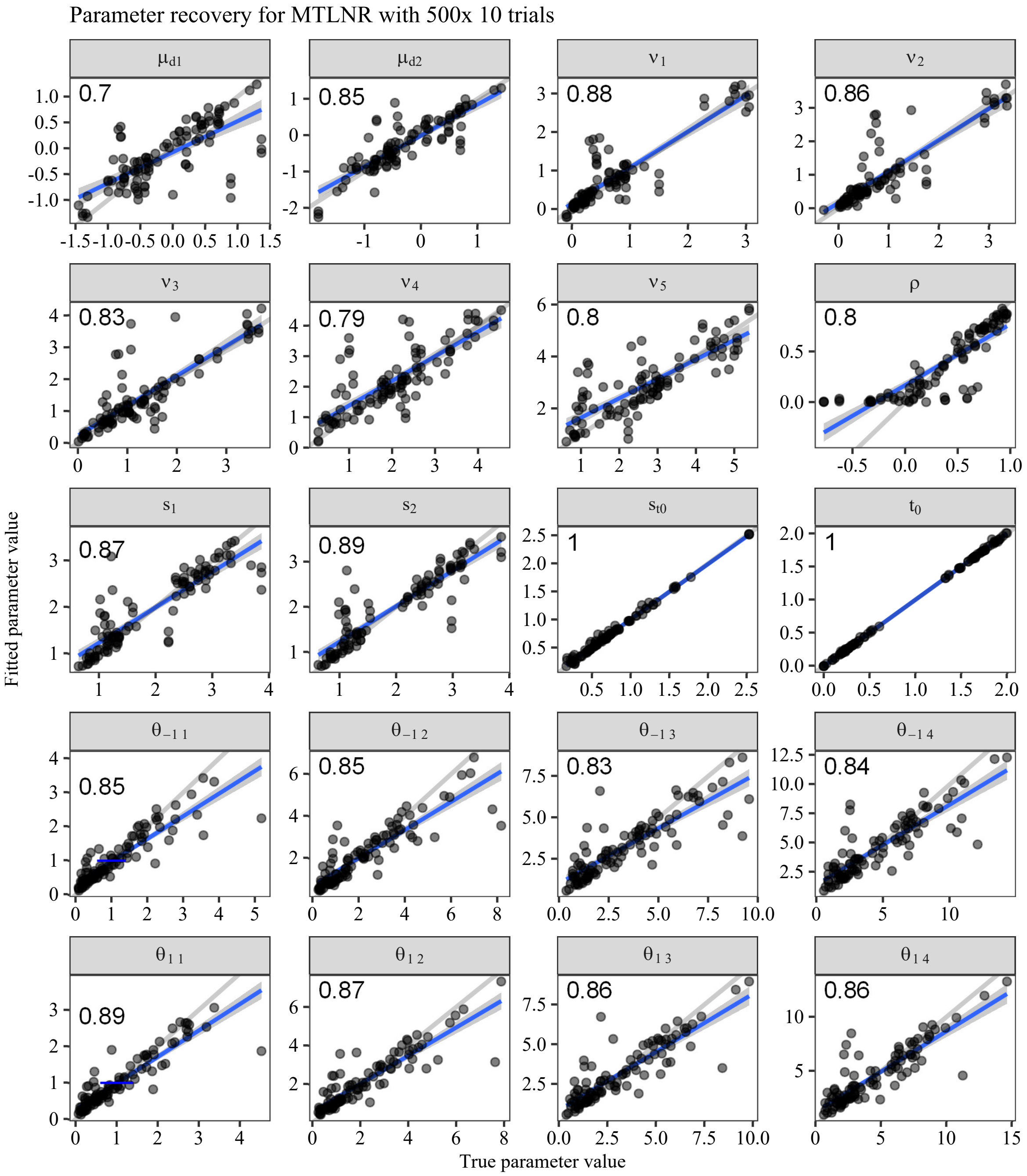

Supplementary Figure 22: Results from parameter recovery analysis for MTLNRwith 500 trials per condition and stimulus identity. Recovered vs. true generative parameters across parameters. Each point represents one simulated parameter and data set. The blue line and shaded area show a linear regression line with 95% confidence band. The grey line shows the identity line. Numbers in the panels show the concordance correlation coefficient for the parameter.

4 Model Recovery

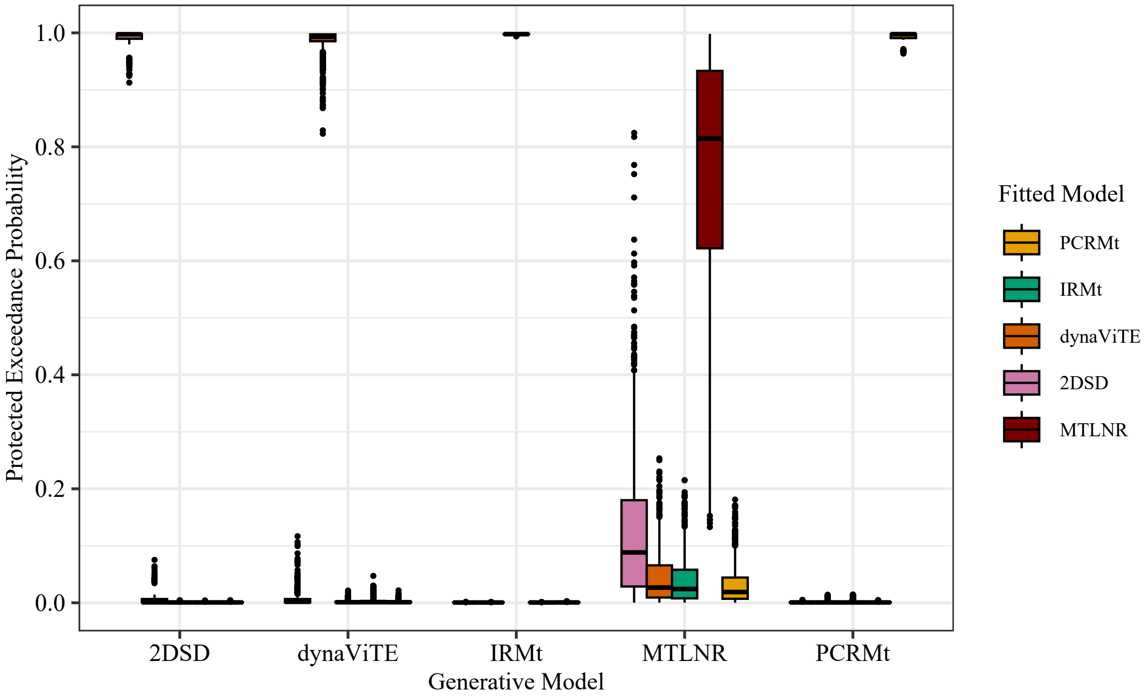

Supplementary Figure 23: Group-level model selection in the model recovery using bootstrapped protected exceedance probabilities (PEP) based on AIC.

References

Hellmann, S., Zehetleitner, M., & Rausch, M. (2023). Simultaneous modeling of choice, confidence, and response time in visual perception. *Psychological review*. https://doi.org/10.1037/rev0000411

Hellmann, S., Zehetleitner, M., & Rausch, M. (2024). Confidence is influenced by evidence accumulation time in dynamical decision models. *Computational Brain & Behavior*, *7*(3), 287–313. https://doi.org/10.1007/s42113-024-00205-9

Lin, L. I. (1989). A concordance correlation coefficient to evaluate reproducibility. *Biometrics*, *45*(1), 255–268.

Ng, L. C. H., Law, F. H. F., Lam, A. M. W., Or, C. C.-F., & Lee, A. L. F. (2021). Metacognitive adaptation revealed in serial dependence of visual confidence judgments. *Journal of Vision*, *21*(9), 2487. https://doi.org/10.1167/jov.21.9.2487

Orchard, E. R., Dakin, S. C., & van Boxtel, J. J. A. (2022). Internal noise measures in coarse and fine motion direction discrimination tasks and the correlation with autism traits. *Journal of Vision*, *22*(10), 19. https://doi.org/10.1167/jov.22.10.19

Reynolds, A., Kvam, P. D., Osth, A. F., & Heathcote, A. (2020). Correlated racing evidence accumulator models. *Journal of Mathematical Psychology*, *96*, 102331. https://doi.org/10.1016/j.jmp.2020.102331

Shekhar, M., & Rahnev, D. (2021). The nature of metacognitive inefficiency in perceptual decision making. *Psychological review*, *128*(1), 45–70. https://doi.org/10.1037/rev0000249
